# Biophysical and proteomic analyses of *Pseudomonas syringae* pv. *tomato* DC3000 extracellular vesicles suggest adaptive functions during plant infection

**DOI:** 10.1128/mbio.03589-22

**Published:** 2023-06-27

**Authors:** Martin Janda, Katarzyna Rybak, Laura Krassini, Chen Meng, Oséias Feitosa-Junior, Egidio Stigliano, Beata Szulc, Jan Sklenar, Frank L.H. Menke, Jacob G. Malone, Andreas Brachmann, Andreas Klingl, Christina Ludwig, Silke Robatzek

**Affiliations:** 1 LMU Munich Biocenter, Ludwig-Maximilian-University of Munich, Munich, Germany; 2 Department of Biochemistry and Microbiology, University of Chemistry and Technology Prague, Prague, Czechia; 3 Faculty of Science, University of South Bohemia in České Budějovice, České Budějovice, Czechia; 4 Bavarian Center for Biomolecular Mass Spectrometry (BayBioMS), Technical University of Munich, Gregor-Mendel-Strasse, Freising, United Kingdom; 5 The Sainsbury Laboratory, Norwich Research Park, Norwich, United Kingdom; 6 John Innes Centre, Norwich Research Park, Norwich, United Kingdom; 7 University of East Anglia, Norwich Research Park, Norwich, United Kingdom; Max Planck Institute for Terrestrial Microbiology, Marburg, Germany; University of California Berkeley, Berkeley, California, USA

**Keywords:** extracellular vesicles, EVs, *Pto *DC3000, proteomics, pattern-triggered immunity, PTI, nanoparticle tracking analysis, NTA, *Arabidopsis thaliana*

## Abstract

Vesiculation is a process employed by Gram-negative bacteria to release extracellular vesicles (EVs) into the environment. EVs from pathogenic bacteria play functions in host immune modulation, elimination of host defenses, and acquisition of nutrients from the host. Here, we observed EV production of the bacterial speck disease causal agent, *Pseudomonas syringae* pv. *tomato* (*Pto*) DC3000, as outer membrane vesicle release. Mass spectrometry identified 369 proteins enriched in *Pto* DC3000 EVs. The EV samples contained known immunomodulatory proteins and could induce plant immune responses mediated by bacterial flagellin. Having identified two biomarkers for EV detection, we provide evidence for *Pto* DC3000 releasing EVs during plant infection. Bioinformatic analysis of the EV-enriched proteins suggests a role for EVs in antibiotic defense and iron acquisition. Thus, our data provide insights into the strategies this pathogen may use to develop in a plant environment.

**IMPORTANCE:**

The release of extracellular vesicles (EVs) into the environment is ubiquitous among bacteria. Vesiculation has been recognized as an important mechanism of bacterial pathogenesis and human disease but is poorly understood in phytopathogenic bacteria. Our research addresses the role of bacterial EVs in plant infection. In this work, we show that the causal agent of bacterial speck disease, *Pseudomonas syringae* pv. *tomato*, produces EVs during plant infection. Our data suggest that EVs may help the bacteria to adapt to environments, e.g., when iron could be limiting such as the plant apoplast, laying the foundation for studying the factors that phytopathogenic bacteria use to thrive in the plant environment.

## INTRODUCTION

Successful colonization of hosts depends on the ability of microbes to defend themselves against host immune responses and acquire nutrients. Bacterial pathogens use macromolecular translocation systems and deliver virulence proteins, so-called effectors, to circumvent host immunity (1). *Pseudomonas syringae* pv. *tomato* (*Pto*) DC3000 is the causal agent of bacterial speck, a common disease that affects tomato production worldwide (2, 3). *Pto* DC3000 is a Gram-negative bacterium that invades through openings in the plant surface and propagates in the apoplast, where it takes up nutrients and proliferates (4 - 6). Plants respond rapidly to colonization by microbes, activating interlinked innate defense strategies (7), which can broadly be categorized into pattern-triggered immunity (PTI) activated by pathogen-associated molecular patterns (PAMPs) and effector-triggered immunity induced upon recognition of virulence factors or their actions (8, 9). Virulence of *Pto* DC3000 largely depends on the type III secretion system and its secreted effectors (10 - 13).

The survival of infectious Gram-negative bacteria is greatly enhanced by releasing extracellular vesicles (EVs), a process widely studied in the context of bacteria pathogenic to humans (14). EVs are cytosol-containing membrane “nano” spheres that provide selection, storage, and protection against degradation of enclosed cargoes in a highly dynamic and environmental cue-responsive manner (14 - 16). EVs can differ in biophysical parameters like size and charge, as well as in cargo composition and biogenesis. Gram-negative bacteria actively form EVs by budding and shedding the outer membrane (OM), producing so-called outer membrane vesicles (OMVs) (17, 18). Outer–inner membrane vesicles (OIMVs) have also been described, involving a different mode of release such as endolysin-triggered cell lysis (19, 20). As insufficient biomarkers are available to convincingly probe their origin, in particular for *P. syringae*, we will collectively refer to these vesicles as EVs.

During infection, bacterial EVs can counteract the effect of antimicrobial peptides (21). They also perform immunomodulatory functions by delivering virulence factors to recipient cells resulting in immune suppression (22), despite having the capacity to activate defenses due to their immunogenic cargoes (22 - 24). Elongation factor Tu (EF-Tu) and lipopolysaccharides (LPS) are abundant components of EVs from *P. syringae*, *Xanthomonas campestris*, *X. oryzae*, and *Xylella fastidiosa* (25 - 28). Both represent PAMPs, with EV-associated EF-Tu shown to activate a prototypic PTI response in a receptor-dependent manner (25, 29). EVs isolated from pathogenic *Pto* DC3000 and the commensal *P. fluorescens* were shown to induce immunity, protecting plants against *Pto* DC3000 infection (30). These studies hint at some contrasting roles that EVs from bacterial phytopathogens could play during plant infection (23).

Here, we used biophysical and biochemical analysis to describe *Pto* DC3000 EVs and to gain insights into their role during infection. Analysis of *Pto* DC3000 cellular, OM and EV proteomes by mass spectrometry identified 369 EV-enriched proteins. The potential function of these proteins was assessed using bioinformatic analysis as well as exploring plant immune responses to EVs and the presence of EVs *in planta* by establishing OprF and β-lactamase as EV biomarkers. These findings expand our understanding of the functions of EVs in bacterial infection of plants.

## RESULTS

### *Pto* DC3000 bacteria vesiculate and produce EVs in culture

**Fig 1:**
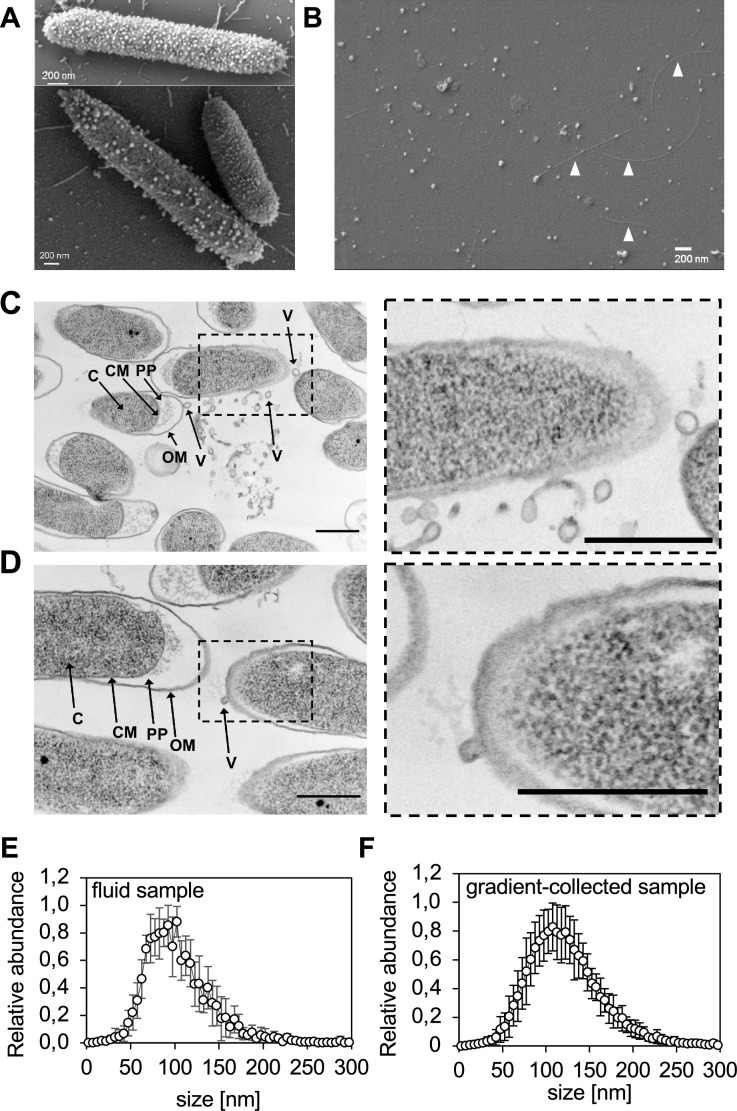
*Pto* DC3000 release extracellular vesicles in the form of OMVs. (**A**) Representative SEM micrographs of planktonic *Pto* DC3000 grown in liquid culture 1.5–2 × 10^9^ cfu/mL (SEM was done in three biological repeats and in all repeats EVs were observed). (**B**) Representative SEM micrograph of gradient-enriched *Pto* DC3000 EVs purified from planktonic culture (1.5–2 × 10^9^ cfu/mL); arrowheads point at filamentous structures co-purifying with the vesicles (SEM was done in three biological repeats). For A and B scale bars = 200 nm. (**C and D**) Left panel shows representative transmission electron microscopy (TEM) micrographs from planktonic *Pto* DC3000 cultures (1.5–2 × 10^9^ cfu/mL). C, cytoplasm; CM, cytoplasmic membrane; PP, periplasm; V, vesicle. For C and D scale bars = 500 nm. (**C**) A lot of smaller and larger vesicles in proximity to cells. It is important to note that the larger vesicle-like structures could also represent debris of dead cells. (**D**) Budding vesicle in the right part of the micrograph. Dashed boxes indicate enlarged regions of the micrographs shown in the right panel. TEM was preformed from three biologically independent bacterial samples. (**E**) Size profile of EVs from *Pto* DC3000 planktonic cultures in fluid samples (3.75–5.5 × 10^9^ cfu/mL); the values represent mean and standard deviations from 13 biologically independent samples. (**F**) Size profile of gradient-collected EVs from planktonic *Pto* DC3000 cultures (1.5–2 × 10^9^ cfu/mL); the values represent mean and standard deviations from 20 biologically independent samples.

We first examined the morphology of *Pto* DC3000 cells grown in liquid cultures by scanning electron microscopy (SEM). The bacteria displayed multiple spherical structures protruding from their cell surfaces, with diameters in the range of 20–120 nm with a median around 35 nm (Fig. 1A; Fig. S1A and B). These vesicle-like structures appeared to be released from the surface, as similarly sized vesicular structures could also be observed in the vicinity of the bacteria.

To determine whether these structures were released, supernatants of *Pto* DC3000 cultures were processed and examined before (fluid sample) and after centrifugation (gradient-collected sample) (Fig. S1C). Density gradient centrifugation is used to separate EVs from other extracellular materials (31). Gradient-collected vesicles were analyzed by SEM, which revealed numerous spherical structures (Fig. 1B). Vesicle diameters ranged between 25 and 170 nm with a median around 80 nm (Fig. S1D). Co-purifying filamentous structures could also be detected (Fig. 1B). Transmission electron microscopy (TEM) analysis-sectioned *Pto* DC3000 samples showed several structures reminiscent of budding vesicles from the bacterial OM (Fig. 1C and D). This suggests that *Pto* DC3000 can produce EVs in the form of OMVs (17, 18).

Nanoparticle tracking analysis (NTA) was used to measure particle size (median diameter and distribution), particle surface charge (mean ζ-potential), and particle number (concentration). In this size analysis, both sample types exhibited a polydisperse-sized population of spherical structures with a diameter ranging from ~50 to 200 nm and median sizes of 100 and 115 nm for fluid samples and gradient-collected samples, respectively (Fig. 1E and F). It is possible that conditions used for SEM and NTA differ in their capacity to hydrate the vesicles and/or that NTA underestimates smaller particles (32).

To determine whether EV production is an active process, EVs were quantified from culture supernatants of *Pto* DC3000 over cultivation time, with increasing particle numbers observed with bacterial density (Fig. S2A and B). Calculation of the amount of EVs produced per bacterium showed that numbers were similar between growth stages (Fig. S2A and B). The median diameter and ζ-potential of EVs were mostly comparable across growth stages, yet differed slightly between the sample types (Fig. S2C and D). Albeit we cannot exclude the possibility that vesicles could be derived, e.g., from exploding cells (20), the vesicles recovered from culture samples appeared to be predominantly produced by bacteria as an active process since the number of dead cells from planktonic cultures was little compared with heat killing (Fig. S2E), which also caused higher particle numbers (Fig. S2F). This is consistent with the observations from TEM (Fig. 1C and D).

### EVs from cultured *Pto* DC3000 are enriched in 369 proteins

**Fig 2:**
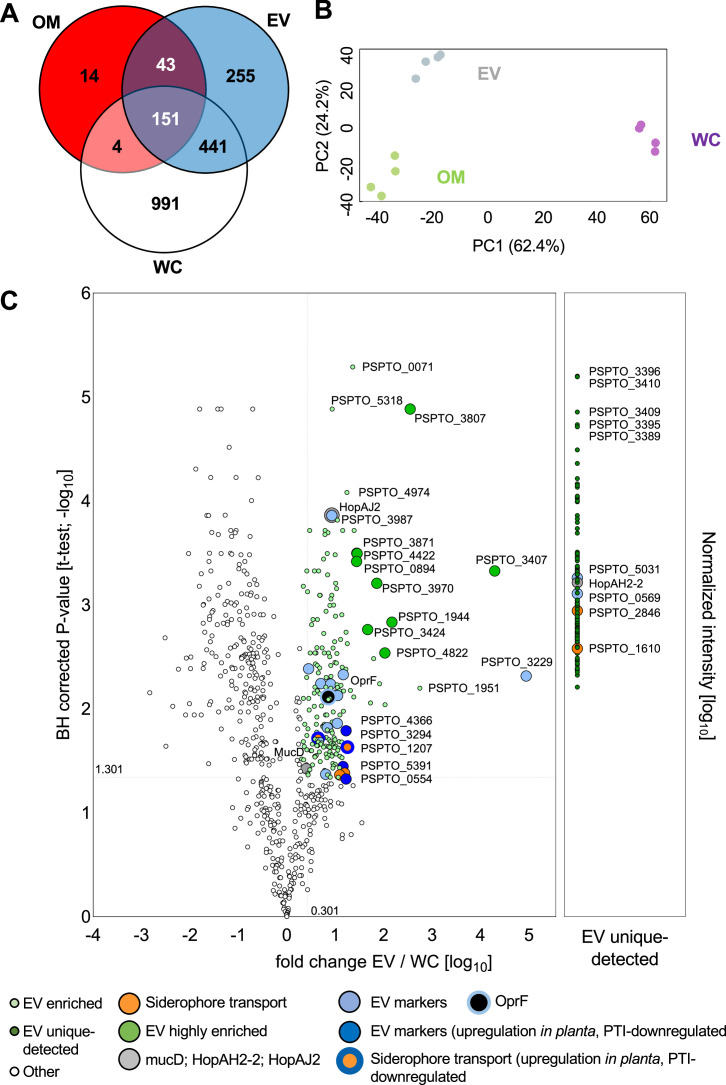
Proteomic analysis identifies 369 proteins enriched in *Pto* DC3000 EVs. (**A**) Comparison of proteins detected in *Pto* DC3000 WC lysate, OM, and EV. Proteomic analysis was done using four biologically independent samples. (**B**) Principal component analysis of identified proteins. (C) Volcano plot comparing EV and WC proteomes. EV-enriched proteins were defined in two categories: (i) fold change EV/WC >2 and false discovery rate < 0.05 (*t*-test); (ii) measured in three replicates in EV but not in WC. In addition, the mean intensity in EV protein needs to be in the top 50% of all proteins, so only high-intensity proteins in EV are selected. Four types of proteins enriched or unique detected in EVs were highlighted: proteins related to virulence; proteins related to siderophore transport; candidate EV biomarkers; and proteins highly enriched in EVs compared with WC.

To gain insights into the biogenesis and functions of *Pto* DC3000 EVs, we characterized the proteome of EVs using liquid chromatography-based tandem mass spectrometry (LC-MS/MS). To this end, we cultivated *Pto* DC3000 in a rich, yet iron-limited medium, allowing for high bacterial growth and thus EV yield as well as considering iron limitation in the leaf apoplast during pathogen infection (25, 33). The *Pto* DC3000 EV-associated proteins were isolated from *Pto* DC3000 cultures by gradient enrichment. In parallel, we determined the proteomes of whole cells (WC) and OM preparations. We detected the highest number of proteins from the WC sample (*n* = 1,587), followed by the EV sample (*n* = 890) and 212 proteins in OM samples (Fig. 2A; Table S1). In total, 2,898 proteins were identified over all samples, of which 1,899 proteins were identified at least in three of the four samples per sample type (WC, EV, or OM). These proteins were taken forward for further analysis (Table S1). Similar protein intensity distributions were obtained for all samples [label-free quantification (LFQ) values were generated by MaxQuant, Fig. S3], and the four replicate measurements per sample type fell into sample clusters on the first and second principal components, suggesting a systematic difference in the proteomes of these three sample types (Fig. 2B). By comparing the proteomes of EV and WC, we identified 369 EV-enriched proteins, consisting of 162 proteins exclusively identified in at least 3 replicates of EV sample (EV unique detected; Fig. 2C) and 207 proteins significantly higher in the EV compared with WC (Fig. 2C; Table S1). Next, we analyzed the proteomics data (i) using computational approaches (bioinformatics and database searches) and (ii) building on current knowledge (EV biogenesis and immunomodulatory activities).

### *Pto* DC3000 EVs are enriched in proteins with predicted roles in transport and antimicrobial peptide resistance

**Fig 3:**
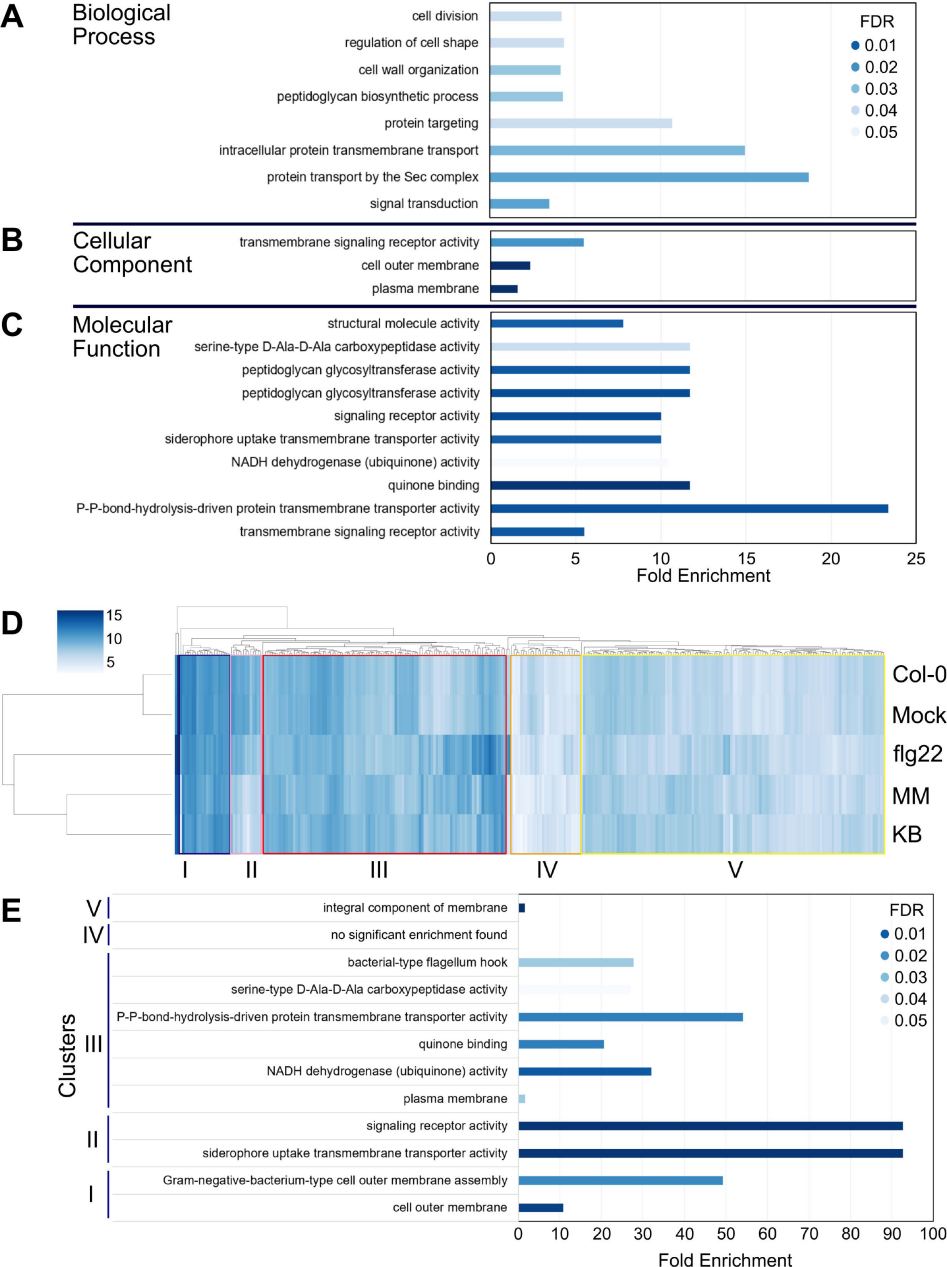
Proteomic composition of *Pto* DC3000 EVs suggests functions in immunomodulation and host adaptation. (**A through **C) Enriched proteins in biological processes (**A**), cellular localization (**B**), and molecular function (**C**). (**D**) Heat map representing transcriptional patterns of the genes coding for EV-enriched proteins. Genes with similar expression patterns are indicated as clusters. Five clusters (I–V) were highlighted. Transcriptome data are derived from reference (33). (**E**) GO terms associated with clusters.

We performed a gene set analysis on the 369 EV-enriched proteins to examine the biological processes, cellular component, and molecular function in which these proteins are involved [from gene ontology (GO)] (34, 35). In total, 20 GO terms were significantly enriched [Fig. 3A through C; false discovery rate (FDR) <0.05; DAVID bioinformatics resources] (36, 37). Eight terms were found in the category “biological process,” out of which, four terms were associated with “cellular processes” related to cell division, shape, and cell wall remodeling. An increasing release of EVs was observed in cells that grow at exponential phase, likely due to an increased turnover of peptidoglycan during cell division (38). Three terms were connected to the general process of “transport,” including transmembrane transport, intracellular transmembrane transport, and protein transport by the Sec complex (Fig. 3A; Table S2). This suggests a specific and/or selective mechanism for the delivery of protein cargoes into EVs. Indeed, classification of the proteins enriched in EVs by putative subcellular localization revealed distinct localization profiles compared with WC and OM proteins. While 66% of WC proteins were cytoplasmic, about half (51%) of the EV-enriched proteins were cytoplasmic membrane associated, with the next largest known class being OM associated (11%) (Fig. S3C). This is consistent with the four terms found in the category “cellular component,” connected to the general compartment “membrane,” including OM and plasma membrane (Fig. 3B). Combining the data from proteomics and TEM, it may suggest that *Pto* DC3000 produces EVs in the form of both OMVs and OIMVs, as previously described for the closely related species *P. aeruginosa* (20). The category “molecular function” was enriched in eight terms, including processes associated with peptidoglycan synthesis also found in the “biological process” category, and siderophore transport (Fig. 3C). Siderophores are secondary metabolites that can sequester iron. Bacteria secrete siderophores under iron-limiting environments, improving iron uptake and thereby contributing to bacterial survival (39). The enrichment of siderophore transport proteins in *Pto* DC3000 EVs suggests that the release of EVs may contribute to the acquisition of iron.

EV yield and likely cargo composition are affected by the environment, in which bacteria grow (25). Having identified proteins enriched in EVs collected from cultured *Pto* DC3000 bacteria, this could limit evidence on the role of EV-enriched proteins during plant infection. If EV-enriched proteins would be involved in infection, we assumed that (i) proteins enriched in EVs from cultured bacteria would be present in bacteria *in planta* and, thus, (ii) genes coding for EV-enriched proteins would be expressed in bacteria *in planta*, and (ii) genes coding for EV-enriched proteins could respond to the plant’s immune status. We, therefore, inspected available *Pto* DC3000 transcriptome data (33).

The expression patterns of genes coding for EV-enriched proteins differed mostly between *Pto* DC3000 cultured *in vitro* (in both minimal and rich media), present *in planta* (in both untreated and mock-treated plants), and present in flg22 immune-induced plants (Fig. 3D). We found five clusters of gene expression patterns across these conditions. Of note, genes in cluster II were upregulated in bacteria in response to *in planta* conditions but downregulated in bacteria from flg22-induced plants. It is thus possible that the proteins encoded by the genes in cluster II are also present at EVs produced by *Pto* DC3000 in untreated and mock-treated plants. Since cluster II is enriched in the GO term “siderophore uptake transmembrane transporter activity” (Fig. 3E; Table 1; Table S2), EVs may play roles in iron acquisition (Fig. 2C, orange labeling).

**TABLE 1:** Protein list of the genes present in cluster II and their predicted localization

Locus tag	Subcellular localization	Product description^*a*^	Other
PSPTO_0577	Unknown	Phage tail sheath subtilisin-like domain-containing protein	
PSPTO_0753	Cytoplasmic membrane	Multidrug efflux MFS transporter	EV unique detected
PSPTO_1207	Outer membrane	TonB-dependent siderophore receptor	EV marker; siderophore transport
PSPTO_1760	Cytoplasmic membrane	HAAAP family serine/threonine permease	
PSPTO_2115	Outer membrane	VacJ family lipoprotein	
PSPTO_3294	Outer membrane	TonB-dependent receptor	EV marker; siderophore transport
PSPTO_3574	Outer membrane	TonB-dependent siderophore receptor	Siderophore transport
PSPTO_4196	Cytoplasmic membrane	Glucose/quinate/shikimate family membrane-bound PQQ-dependent dehydrogenase	
PSPTO_4452	Cytoplasmic	LPS export ABC transporter ATP-binding protein	
PSPTO_4883	Cytoplasmic membrane	PepSY domain-containing protein	
PSPTO_4931	Cytoplasmic membrane	Hypothetical protein	EV unique detected
PSPTO_5391	Outer membrane	OprD family porin	EV marker
PSPTO_5603	Cytoplasmic membrane	F0F1 ATP synthase subunit B	EV “core”

^*a*^ MFS = Major Facilitator Superfamily; PQQ = pyrroloquinoline quinone; ABC = ATP-binding cassette.

Cluster III contains genes that were similarly expressed in bacteria grown *in vitro* and bacteria from flg22-induced plants but differed in their expression in response to *in planta* conditions (Fig. 3D). This cluster is associated, e.g., with the GO term “bacterial-type flagellum hook” (Fig. 3E), which has been described in the biogenesis of EVs (40). Also, cluster III is associated with the GO term “serine-type D-Ala-D-Ala carboxypeptidase activity,” which has a cross-reference with the term “Penicillin-binding protein 2” in the InterPro database of protein families. It is worth mentioning that genes PSPTO_3987 and PSPTO_4977 both annotated with the “β-lactam resistance” function are also present in cluster III, although not assigned in the above-mentioned GO term.

### Purified *Pto* DC3000 EV samples have immunomodulatory activities

**Fig 4:**
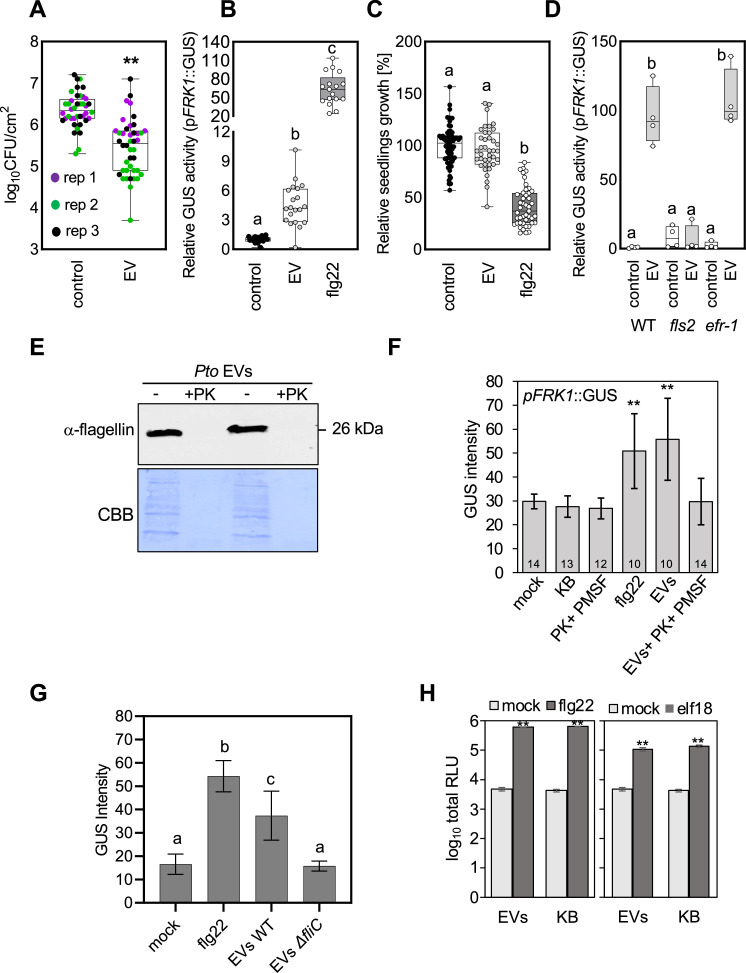
Immunogenic activities of *Pto* DC3000 EVs are mostly dependent on flagellin-induced responses. (**A**) *Pto* DC3000 growth (cfu) after infection into leaves of *A. thaliana* without and with EV pretreatment (24 hours) at 3 dpi (control = 0.02 mM EDTA). Three biological repeats, each consisting of 12 independent samples were performed. The dots with the same color represent independent samples from one biological repeat. (**B**) Quantification of *pFRK1*::GUS activity in seedlings incubated without (*n* = 20) and with EVs (*n* = 20) or with 100 nM flg22 (*n* = 17) for 18 hours. The graph represents independent samples from four biological repeats. (**C**) Fresh weight of seedlings grown without and with EVs for 8 days. For control *n* = 69; for 100 nM flg22 treatment *n* = 44, and EV treatment *n* = 39 of independent samples. The experiment was repeated in six biological repeats with similar results. (**D**) Relative *FRK1* gene expression in seedlings of the indicated genotypes incubated without and with EVs for 5 hours (control = 0.02 mM EDTA), four independent samples were used for each variant. The experiment was repeated in two biological repeats with similar results. For A, B, C, and D were used EVs for treatments in concentration ≈1.10^10^/mL. The boxplots extend from 25th to 75th percentiles, whiskers go down to the minimal value and up to the maximal value, and the line in the middle of the box represents the median. Asterisks indicate statistical significances (two-tailed Welsch’s *t*-test; *P* < 0.01) in A; different letters indicate significant differences (Welsch’s analysis of variance with Dunnett’s T3 multiple comparisons post hoc test; *P* < 0.05) in B, C, and D. (**E**) Immunoblot of flagellin in *Pto* DC3000 EVs without and with proteinase K treatment. Flagellin antibodies detect bands in untreated gradient-collected EVs but not proteinase K-treated EVs. Coomassie brilliant blue (CBB) shows protein loading, evidently significantly reduced by proteinase K treatment. (**F**) Quantification of *pFRK1*::GUS signals in seedlings incubated with gradient-collected *Pto* DC3000 EVs (concentration ≈1.10^10^) without and with proteinase K treatment (PK+ PMSF [phenylmethylsulfonyl fluoride]), 100 nM flg22, and particles (concentration ≈1.10^10^) of unconditioned KB medium for 24 hours. The seedling number *n* is shown in the bars; the bars represent average, and error bars are SD. Asterisks indicate significant differences based on t-test analysis. The experiment was repeated at least twice with similar results. (**G**) Quantification of *pFRK1*::GUS signals in seedlings (12 days old) incubated with EVs (concentration ranging from 0.75 to 2.5 × 10^10^/ mL) from *Pto* DC3000 wild-type (WT) and Δ*fliC* mutants medium for 18 hours. The bars represent mean, and error bars are SD from *n* = 10 independent samples. The experiment was done in two biological repeats with similar results. (**H**) Quantification of PAMP-induced reactive oxygen species (ROS) in leaves pre-treated without and with EVs (concentration ≈1.10^10^) for 24 hours. The bars represent mean, and error bars are SD from *n* = 10 leaf discs. Asterisks indicate significant differences based on t-test analysis. ROS experiments were repeated at least twice with similar results.

Given the presence of flagellin in our *Pto* DC3000 EV samples, we next examined the ability of the *Pto* DC3000 EVs to modulate the outcome of bacterial infection. We pretreated *Arabidopsis thaliana* leaves with *Pto* DC3000 EVs, which limited the growth of subsequently infected *Pto* DC3000 bacteria *in planta* (Fig. 4A). Thus, the immunogenic activity of *Pto* DC3000 EVs is sufficient to restrict bacterial colonization, consistent with recent observations (30). In agreement, seedlings treated with purified EVs showed induction of *pFRK1*::GUS expression, albeit lower when compared with treatments with flg22 (Fig. 4B; Fig. S4A). We also tested whether treatment with *Pto* DC3000 EVs could arrest seedling growth, a prototypic PTI response of plants to continual PAMP stimulation (41). We observed no significant growth reduction in this experiment (Fig. 4C).

Six flagella-associated proteins were enriched in *Pto* DC3000 EVs, of which flagellin was more than twofold enriched relative to the WC proteome (Table S1). Therefore, to determine the pathway by which the *Pto* DC3000 EVs trigger immune responses, we treated *A. thaliana* mutants of the FLAGELLIN SENSING 2 (FLS2) and EF-Tu receptor (EFR) immune receptors responsible for recognition of flg22 and elf18, respectively (42). The *Pto* DC3000 EVs triggered *FRK1* gene expression in wild-type (WT) and *efr-1* mutants to similar levels (Fig. 4D). No *FRK1* induction was observed in *fls2* mutants. Thus, the EV samples isolated from *Pto* DC3000 cultures must contain bacterial flagellin as the immunogenic molecule.

Notably, SEM analysis of gradient-collected EV samples showed the co-purification of filament-like structures (Fig. 1B), which could represent detached bacterial flagellar or pili. Since flagellin could not be detected in proteinase K-treated EVs and proteinase K-treated EVs did not significantly induce *pFRK1*::GUS expression (Fig. 4E and F; Fig. S4B), taken together, it is possible that flagellin is a co-purifying immunogenic molecule present in *Pto* DC3000 EV samples and recognized in *A. thaliana*. This is consistent with the observation that EVs purified from the *Pto* DC3000 Δ*fliC* mutant did not significantly induce *pFRK1*::GUS expression (Fig. 4G; Fig. S4C). Considering co-purifying flagellin as the major immunogenic molecule in *Pto* DC3000 EV samples, its amount might be insufficient to repress seedling growth over time.

The EV-enriched proteome included proteins related to virulence (Fig. 2C; Table S1), such as MucD (PSPTO_4221) (43), HopAJ2 (PSPTO_4817) (44), and HopAH2-2 (PSPTO_3293) (45, 46). A major function of virulence proteins is the suppression of PTI (47). Recently, the integration of *X. campestris* pv. *campestris* OMVs into plant plasma membranes was observed, which might suggest that vesicle cargoes such as virulence proteins could be discharged into plant cells (48). To test whether *Pto* DC3000 EVs could modulate a prototypic PTI response, we pretreated leaves with EVs from cultured bacteria 24 hours before eliciting an ROS burst with the immunogenic peptides flg22 from bacterial flagellin and elf18 from EF-Tu. EV pretreatments neither significantly reduced nor increased the PAMP-induced ROS production (Fig. 4H). This suggests that under the tested conditions, *Pto* DC3000 EVs are not predominantly involved in inhibiting and/or further enhancing the PAMP-induced ROS responses.

### *Pto* DC3000 bacteria produce EVs *in planta*

The observation that EVs collected from *Pto* DC3000 cultures may not play major roles in host immune modulation raises the question whether *Pto* DC3000 releases EVs during plant infection. To address this, apoplastic fluids were recovered from *Pto* DC3000-infected and control-treated *A. thaliana* leaf tissues at different time points and filtered to remove bacteria.

SEM analysis of apoplastic fluids from control and infected leaves revealed the presence of vesicle-like particles and tiny particles, the latter could resemble ribosomes and/or larger protein complexes (Fig. S5A and B). In apoplastic fluids from infected leaves, we additionally observed structures reminiscent of pili and/or flagellar (Fig. S5B).

**Fig 5:**
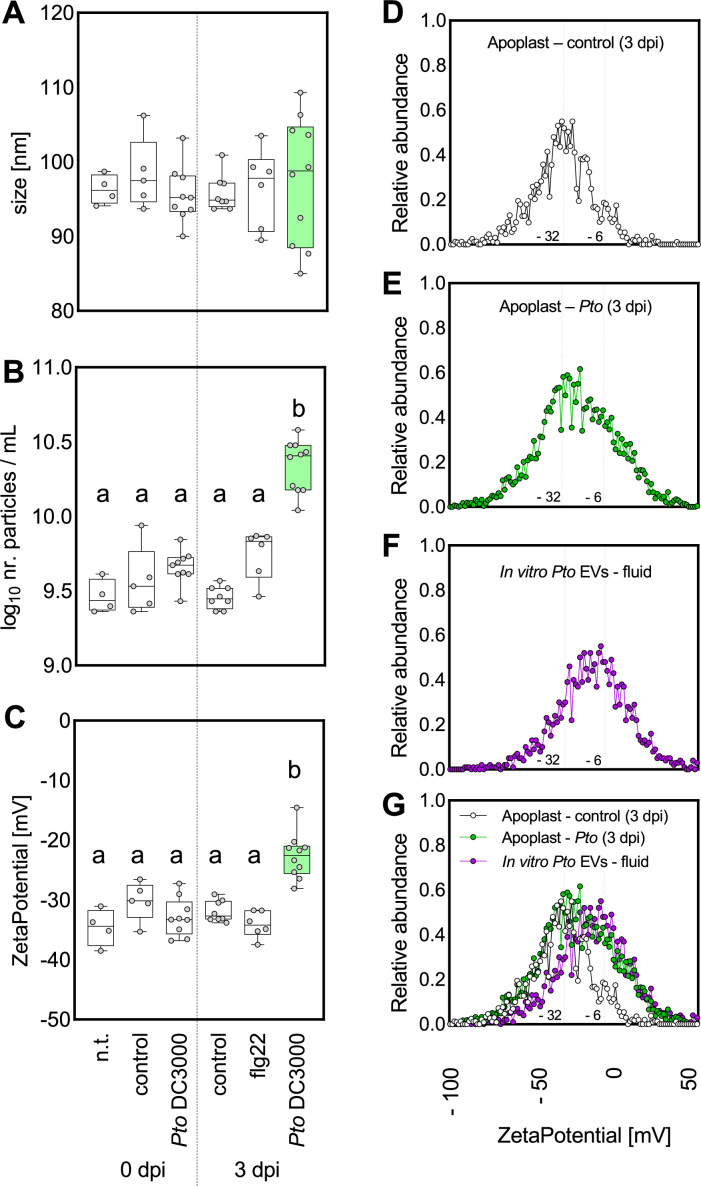
*Pto* DC3000 release EVs *in planta*. Size, concentration, and charge measurements of apoplastic fluids from *A. thaliana* plants (5–6 weeks old) infected with *Pto* DC3000. (**A**) Size of the particles. (**B**) Particle concentration in apoplastic fluids. (**C**) ζ-Potential of the particles. For A–C, the variants represent: n.t., nontreatment (*n* = 4); control, 10 mM MgCl_2_ (0 dpi, *n* = 5; 3 dpi, *n* = 8); *Pto* DC3000 (OD_600_ = 0.0006) (0 dpi, *n* = 9; 3 dpi, *n* = 10); 100 nM flg22 (3 dpi, *n* = 6). Experiments A–C were performed in three biological repeats with similar results. (**D–G**) The profile of ζ-potential for particles detected in *Arabidopsis* apoplast treated with MgCl_2_ (control; **D**) for 3 days, with *Pto* DC3000 (*Pto*; OD_600_ = 0.0006; (**E**) for 3 days and with EVs from *Pto* DC3000 grown in culture (fluid; **F**). The dots represent the mean from 8 (D and G, white), 10 (E and G, green), and 13 (F and G, purple) biologically independent samples. The boxplots extend from 25th to 75th percentiles, whiskers go down to the minimal value and up to the maximal value, and the line in the middle of the box respresents the median. The dots represent values of independent samples. Different letters indicate significant differences (one-way analysis of variance with Tukey post hoc test; *P* < 0.05). The green color is highlighting the particles from *Pto* DC3000-infected plants (3 dpi).

The size of apoplastic fluid vesicle-like particles determined by both NTA and SEM did not significantly differ between control and infected leaves (Fig. 5A; Fig. S5C). Particle abundance increased upon infection with *Pto* DC3000 (Fig. 5B), consistent with previous findings (49). Increased particle abundance correlated with both bacterial infection time and titers (Fig. 5B; Fig. S6A and B). We also analyzed EVs from the apoplastic fluids of plants that were co-treated with 100 nM flg22 and *Pto* DC3000. Particle numbers were lower than those recovered from *Pto* DC3000 infection only, consistent with induced plant resistance and not significantly different from plants only stimulated with flg22 (Fig. 5C; Fig. S6C). Taken together, comparing the particle profiles of fluids isolated from *Pto* DC3000-infected plants with flg22 immune-stimulated plants, both the higher particle number and the polydisperse particle size hint at bacterial-derived EVs present in the apoplast of infected *A. thaliana*.

Since *Pto* DC3000 (fluid sample) and *A. thaliana* (apoplastic fluid samples) EVs did not significantly differ in diameter (Fig. 1E and 5A), we focused on the charge of EVs, reflecting the different surface composition of bacterial (prokaryotic) and plant-derived (eukaryotic) EVs. Evaluation of the mean ζ-potential identified significantly less negatively charged EVs recovered from apoplastic fluids of *Pto* DC3000-infected plants at 3 days post-infection (dpi) compared with control treatments and earlier time points (Fig. 5C). This time point correlated with *in planta* bacterial proliferation and depended on bacterial inoculum (Fig. S6A and B). Plotting the relative particle abundance over particle charge, the ζ-potential profiles of EVs recovered from apoplastic fluids of untreated, control-treated, and flg22-treated *A. thaliana* identified major peaks around −32 mV (Fig. 5D; Fig. S6J). By contrast, the ζ-potential profile of EVs recovered from apoplastic fluids of *Pto* DC3000-infected *A. thaliana* had a broader distribution with a similar major peak around −32 mV and an additional shoulder around −10 mV (Fig. 5E; Fig. S6K). Comparison of the different ζ-potential profiles revealed similarities of the major −32 mV peak across all plant samples, likely representing a plant-derived EV pool. Notably, the shoulder around −10 mV detected from apoplastic fluids of *Pto* DC3000-infected plant samples showed an overlay with the ζ-potential profile of EV recovered from *Pto* DC3000 cultures (fluid samples), with a peak from −20 mV to 0 mV (Fig. 5F and G). This could, therefore, represent a bacterial-derived EV pool. Since the ζ-potential profiles of EVs recovered from apoplastic fluids of flg22-treated *A. thaliana* did not differ between untreated and control-treated leaves (Fig. S6J), we found no evidence that plant EVs modulate their surface charge during infection.

### *Pto* DC3000 EV-associated proteins are detected during plant infection

We next aimed to identify EV-associated proteins that could be used as markers for *Pto* DC3000 EVs *in planta*. To this end, we addressed whether the protein composition of EVs from *Pto* DC3000 and EVs from related bacteria shares similarities. We focused on three published *P. aeruginosa* PAO1 EV proteomes since a number of EV proteomes have been reported from *P. aeruginosa* (50 - 52). We found that 103 proteins were identified in the EV proteomes across the three reports (50 - 52). Of the 103 shared EV proteins from PAO1, we could identify 100 orthologous proteins encoded in the *Pto* DC3000 genome, and 44 proteins were enriched in *Pto* DC3000 EVs (Table 2; Table S1). We refer to these as the EV “core.” These proteins were highly enriched in localization to the OM (44%) and cytoplasmic membrane (26%) (Fig. S7A), consistent with EVs released in the form of OMVs. From these 44 proteins, 20 were putative OM-localized proteins and thus represent good candidate biomarkers for the detection of EVs (Table S1; Fig. 2C, blue labeling). To gain insights into the expression of the EV “core” encoding genes during infection, we searched available transcriptome data (33), which revealed two clusters of generally lower or higher expression levels across the conditions (Fig. S7B). Of the genes coding for EV “core” proteins, a majority encoding OM-localized and cytoplasmic proteins were upregulated *in planta* (Table S1), which suggests their presence during bacterial infection.

**TABLE 2:** List of candidate EV marker proteins

Locus tag	UniProt ID	Subcellular localization	Name	Other
PSPTO_0554	Q88A43	Outer membrane	Organic solvent tolerance protein	
PSPTO_0569	Q88A28	Outer membrane	Autotransporting lipase, GDSL family	EV unique detected
PSPTO_1207	Q887S9	Outer membrane	Iron(III) dicitrate transport protein fecA	Siderophore transport
PSPTO_1296	Q887J6	Outer membrane	Porin B	
PSPTO_1437	Q886Y7	Outer membrane	Lysyl-tRNA synthetase	
PSPTO_1542	Q886N5	Outer membrane	Outer membrane protein	
PSPTO_1720	Q885W1	Outer membrane	Outer membrane protein	
PSPTO_2272	Q883S8	Outer membrane	Outer membrane lipoprotein OprI	
PSPTO_2299	Q883Q1	Outer membrane	Outer membrane porin OprF	
PSPTO_3229	Q880E1	Outer membrane	Filamentous hemagglutinin, intein containing	
PSPTO_3294	Q87ZX8	Outer membrane	TonB-dependent siderophore receptor	Siderophore transport
PSPTO_3971	Q87Y41	Outer membrane	Peptidoglycan-associated lipoprotein	
PSPTO_3987	Q87Y25	Outer membrane	Porin D	beta-Lactam resistance
PSPTO_4115	Q87XR1	Outer membrane	Lipoprotein SlyB	
PSPTO_4366	Q87X24	Outer membrane	Iron-regulated protein A	
PSPTO_4839	Q87VU6	Outer membrane	Hypothetical protein	
PSPTO_4940	Q87VJ6	Outer membrane	HflK protein	
PSPTO_4977	Q87VG0	Outer membrane	Outer membrane efflux protein TolC	Bacterial secretion system
				
PSPTO_5031	Q87VA8	Outer membrane	Type IV pilus biogenesis protein PilJ	beta-Lactam resistance; EV unique detected
PSPTO_5391	Q87UB4	Outer membrane	Outer membrane porin, OprD family	

**Fig 6:**
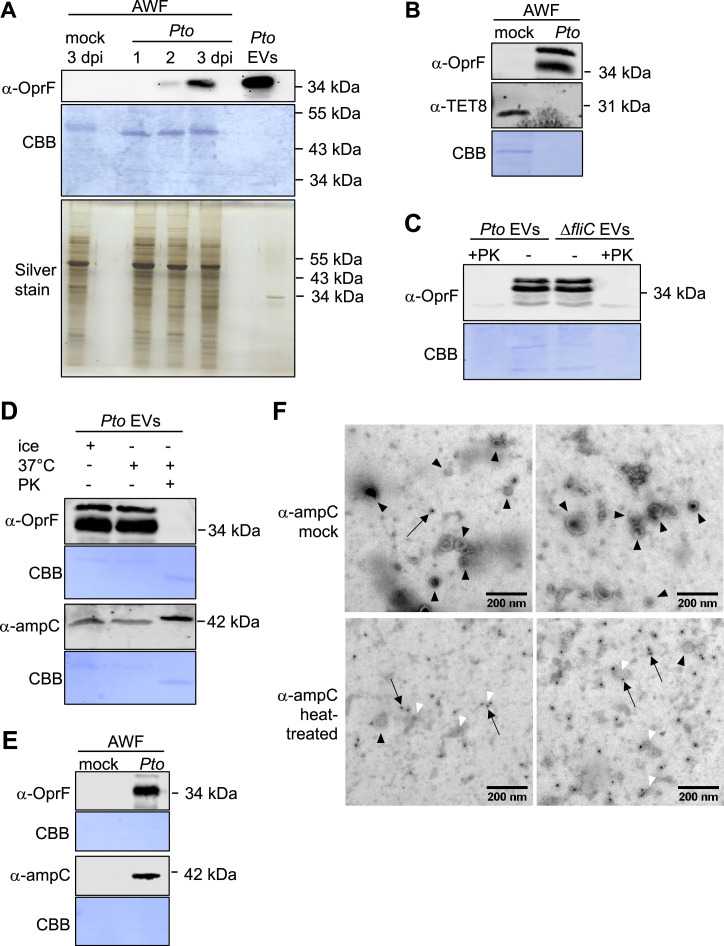
*Pto* DC3000 EV-enriched proteins can be detected in EV samples from filtered apoplastic wash fluids of infected plants. (**A**) OprF antibodies detect bands in apoplastic fluids (AWF) from *A. thaliana* infected with *Pto* DC3000 and in gradient-collected EVs. Coomassie brilliant blue (CBB) and silver staining show protein loading, evidently lower in gradient-collected EV samples. Similar results were observed in at least three independent experiments. (**B**) OprF-positive *Pto* DC3000 EVs are detected in apoplastic fluids containing TET8-positive EVs from infected *A. thaliana*. (**C**) Gradient-collected EVs from *Pto* DC3000 WT and Δ*fliC* mutants show proteinase K (PK)-sensitive detection of OprF. (**D** and **E**) β-Lactamase antibodies ampC detect bands in gradient-collected EVs independent of PK treatment (**D**) and in apoplastic fluids from *A. thaliana* infected with *Pto* DC3000 at 3 dpi (**E**). Here, the blot was incubated with α-ampC antibodies o/n at 4°C. (B through E) CBB shows protein loading, evidently reduced by PK treatment. Similar results were observed in at least three independent experiments. (**F**) Immunogold staining of gradient-collected *Pto* DC3000 EVs using ampC antibodies. Several intact EVs (black arrowheads) were present in mock-treated samples, mostly not immunogold labeled (black arrow). Only in rare cases, gold particles were found at membranous debris. In heat-treated samples, a large amount of membraneous structures (gray areas, white arrowheads) were observed, likely due to the disruption of EVs by the heat treatment. Here, immunogold labeling was observed at the membranous debris (some gold particles are indicated with white arrows). Two representative images are shown for each condition.

One of the predicted EV markers is OprF (Fig. 2C, black labeling), which we used for immunodetection of *Pto* DC3000 EVs *in planta* and purified from *Pto* DC3000 cultures. OprF is a porin integral to the OM and found in EVs of *P. aeruginosa* (53). Using anti-OprF antibodies, we identified specific bands in filtered apoplastic fluids of *A. thaliana* leaves infected with *Pto* DC3000 at 2 and 3 dpi but not in control-treated plants (Fig. 6A). In combination with using anti-TET8 (anti-TETRASPANIN 8) antibodies, we could confirm that the apoplastic fluids of control and infected leaves contain both plant-derived and bacterial EVs (Fig. 6B). OprF-positive EVs were also detected from the *Pto* DC3000 Δ*fliC* mutant (Fig. 6C), confirming successful purification of EVs from this strain. However, OprF could not be detected in proteinase K-treated EVs (Fig. 6C). In *P. aeruginosa*, OprF is a general porin of the OM (54) and, consistently, we detected OprF also in the proteome of the OM samples (Table S1). It is thus possible that the immunogenic epitope recognized by the anti-OprF antibodies is located at the external side of the EVs, making it sensitive to proteinase K treatment. It is currently emerging that the EV surface corona plays roles in EV functions (55), and it is possible that OprF might be part of the *Pto* DC3000 EV corona.

We next aimed to identify an EV-enriched protein localized to the EV lumen and explored available antibodies. For example, EVs of *Stenotrophomonas maltophilia* contained β-lactamase (56). Given that two genes annotated with the “β-lactam resistance” function were present in cluster III and although not detected in our proteome samples, we reasoned that β-lactamase could be present in *Pto* DC3000 EVs. Using anti-β-lactamase antibodies, we identified a specific band in gradient-collected EV samples of cultured *Pto* DC3000, which was also detected after proteinase K treatment (Fig. 6D; Fig. S7C). In addition, a band could be revealed in apoplastic fluids collected from *Pto* DC3000-infected leaves, suggesting the presence of β-lactamase-positive *Pto* DC3000 EVs in these samples (Fig. 6E; Fig. S7C). We explored immuno-negative staining to gain further insights into the localization of β-lactamase in *Pto* DC3000 EVs (Fig. 6F). In mock samples, several intact vesicles were found yet without significant immunogold labeling. Only in rare cases, gold particles were observed at membranous debris. By contrast, after breaking up the vesicles by heat treatment, the membraneous structures showed clear immunogold labeling as several gold particles were observed at these structures. This suggests that β-lactamase was more accessible to ampC immunogold labeling when EVs were disrupted, consistent with β-lactamase present in the lumen of *Pto* DC3000 EVs. Having identified two possible markers for *Pto* DC3000 EVs, membrane-bound OprF and soluble β-lactamase located at the external side and the lumen of EVs, respectively, these data provide additional evidence that *Pto* DC3000 releases EVs *in planta* during infection.

## DISCUSSION

A range of activities has been associated with bacterial EVs during infection. This includes modulation of host immunity (PAMPs, effectors, and bacterial cell wall remodeling), elimination of host defenses (antibiotic tolerance and decoys), and acquisition of nutrients from the host (metal ions) (57). In this study, we used a proteomics approach aiming to gain insights into the presence and role of *Pto* DC3000 EVs during plant infection.

PTI responses to *Pto* DC3000 involve recognition by FLS2, EFR, and LIPO-OLIGOSACCHARIDE-SPECIFIC REDUCED ELICITATION (LORE), which detect immunogenic flg22, elf18, and 3-OH-FAs, a fatty acid co-purifying with LPS (58). EVs from bacterial phytopathogens are enriched in EF-Tu and LPS (25, 26, 28), suggesting the presence of elf18 and 3-OH-FAs. Bahar et al. demonstrated that BRI1-ASSOCIATED KINASE 1 (BAK1) and SUPPRESSOR OF BIR 1 (SOBIR1), interacting co-receptors of PRRs (pattern recognition receptors), mediate the immunogenic perception of EVs from *X. campestris* pv. *campestris* (25). We show that vesicle samples from *Pto* DC3000 elicit immune responses that are dependent on FLS2 and the presence of flagellin in EV samples (Fig. 4 thorugh G). Since flagella proteins such as FliC have a specific affinity for EVs and are involved in EV production in *Escherichia coli* (40), they could be considered external EV cargoes. However, filamentous structures were observed in SEM analysis (Fig. 1B). It is, therefore, possible that flagella co-purify with the *Pto* DC3000 EV samples despite depleting extracellular components from EV samples by density gradient centrifugation. Contamination of flagella in EVs was reported to contribute to the detection of FliC in EVs from *P. aeruginosa* (59).

In contrast to our results, McMillan et al. reported significant seedling growth repression in response to *Pto* DC3000 EVs (30). This disparity in results may be due to several factors, including differences in the growth conditions of both the bacterial cultures and the *A. thaliana* seedlings, the type of biochemical isolation of EVs and vesicle dose, e.g., resulting in different amounts of co-purifying flagella. The stronger protective immune response observed by McMillan et al. may also be due to such differences in experimental procedures (30). Importantly, EVs purified from *Pto* DC3000 *fliC* mutant bacteria did not significantly induce defense gene expression (Fig. 4G). This non-significant immunogenicity of *Pto* DC3000 EVs would be congruous with Pto DC3000 releasing EVs in favor of plant infection.

The plant’s apoplast, which is the niche colonized by *Pto* DC3000, represents an environment where bacteria are challenged with plant defense molecules, competition with members of the microbiome, and acquisition of nutrients (33). Bacteria respond to environmental stress with the production of EVs, which allows for cell surface remodeling, secretion of degraded and damaged cargo, and uptake of nutrients in bacterial communities, e.g., by packaging transporters in EVs (14, 20, 60). The hypothesis that *Pto* DC3000 may use EVs to adapt to the host environment is evidenced by our finding that *Pto* DC3000 EVs contain β-lactamase (Fig. 6D). Several studies demonstrated that EVs can improve bacterial survival during antibiotic exposure. *S. maltophilia* produced more EVs upon treatment with the β-lactam antibiotic imipenem (56, 61). Its EVs contained β-lactamase and increased *S. maltophilia* survival in the presence of antibiotics (56). Plants defend infection by upregulation of many defense-related genes, including genes coding for antimicrobial peptides (62). Likewise, the host microbiome protects plants against infectious pathogens (63). It is possible that *Pto* DC3000 produces EVs to counter the action of plant- and microbe-derived antimicrobial peptides, i.e., its EVs could improve antimicrobial resistance of *Pto* DC3000 (64, 65).

Proteins involved in siderophore transport were enriched in the EVs from cultured bacteria (Fig. 2C). Siderophores are important virulence factors of bacterial pathogens, directly competing for iron with the host (66). Since the ability of *Pto* DC3000 for iron acquisition is correlated with its growth *in planta* (33), siderophores and their transport are likely to play roles in *Pto* DC3000 infection success. Interestingly, the expression of genes coding for all siderophore transport proteins enriched in EVs was upregulated *in planta* compared with *in vitro* conditions as well as downregulated upon induction of PTI (Fig. 3D). Thus, regulation of siderophore transport proteins can be considered as an adaptive response of *Pto* DC3000 to iron/metal ion availability, and secretion into EVs may allow improved acquisition of iron, analogous to EV secretion of the siderophore mycobactin in *Mycobacterium tuberculosis* (67). In addition, the ability of siderophore uptake into EVs may prevent activation of immunity as siderophores can trigger immune responses (66). Taken together, we propose that *Pto* DC3000 produces EVs to improve its growth ability both in culture and *in planta*. Having identified two markers for *Pto* DC3000 EVs, membrane-bound OprF detected as external EV cargo and β-lactamase as luminal EV cargo, future studies to determine the composition of EVs *in planta* and track their interaction with plant cells are now becoming possible.

## MATERIALS AND METHODS

### Bacterial strains and growth

*Pto* DC3000 used in this study was routinely cultured at 28°C in King’s B (KB) medium containing 50 µg/mL rifampicin at 180 rpm and on plates with 1% agar without agitation. Planktonic growth was performed in 500 mL cultures, and growth rates were measured over time as OD_600_. *Pto* DC3000 Δ*fliC* was cultivated at 28°C in KB medium containing 50 µg/mL rifampicin and 5 µg/mL chloramphenicol at 180 rpm (68).

### Plant material and growth conditions

*A. thaliana* ecotype Columbia (Col-0), *pFRK1*::GUS (69), *fls2c* (42), and *efr-1* (70) mutants were used in this study. For bacterial infections and ROS assays, Col-0 plants were soil grown at 21°C–22°C and 8-hour photoperiod. For β-glucuronidase (GUS) assays, RT-qPCR (reverse transcription-quantitative polymerase chain reaction) analysis, and induced growth arrest, seedlings were sterile grown on Murashige and Skoog (MS) plates supplemented with 1% sucrose and 1.5% gelrite (Duchefa, the Netherlands) pH 5.8 for 4 days (after 2–4 days stratification in the dark at 4°C), then transferred to 96-well plates containing 150 µL 1/2 MS medium supplemented with 1% sucrose per well and grown for 11–12 days at 22°C and 16-hour photoperiod (120–150 μE·m^−2^·s^−1^).

### Extraction and purification of bacterial EVs

EVs were routinely isolated from planktonic cultures across growth phases (Fig. S8A). The starting inoculum was OD_600_ = 0.01. Samples were taken according to incubation time, evidently slightly differing in the then measured OD_600_, and therefore, we indicate a range of colony-forming units per milliliter bacterial density, from which EVs were collected. One hundred milliliters of planktonic bacteria (grown in liquid cultures) and 10 mL of biofilm bacteria (grown on plates, collected, and resuspended), respectively, were pelleted at 4,500 × *g* for 2 × 20 minutes, the supernatant was decanted and passed through a 0.22-µm membrane (fluid samples; Fig. S1C). Particles were pelleted from the cell-free supernatant at 100,000 × *g* for 1.5 hours. The pellet was resuspended in 1.7 mL 1 mM EDTA and loaded on sucrose density step-gradient (1.7 mL of sucrose 25%, 35%, 45%, 50%, and 55%) and centrifuged at 160,000 × *g* for 18 hours. Two milliliter samples were collected from each of the sucrose density steps and diluted with 1 mM EDTA to 30 mL. Particles were pelleted at 100,000 × *g* for 2 hours, and the pellets were each resuspended in 0.16 mL 1 mM EDTA (gradient-collected samples; Fig. S1C). EV samples were immediately frozen in liquid nitrogen. Since most EVs migrated to the 55% density fraction (Fig. S8B), we then collected EVs across fractions 3–5, which were less variable in ζ-potential, and size compared to fractions 1 and 2 (Fig. S8C and D).

### Extraction of leaf apoplastic fluids

Apoplastic fluids were collected from leaves of 6–7-week-old plants (Col-0 WT, mock treated, and infected with WT *Pto* DC3000). Rosettes of 22–29 plants were vacuum infiltrated with particle-free 1 mM EDTA. After removing the excess buffer, infiltrated leaves were placed into 20 mL syringes and centrifuged in 50 mL conical tubes at 900 × *g* for 20 minutes at 4°C. The resulting apoplastic wash was passed through a 0.22-µm membrane (apoplastic fluid samples). To confirm the successful filtering of apoplastic fluids, 5 µL was incubated on LB plates containing 50 µg/mL rifampicin. No *Pto* DC3000 colonies were detected after 3 days. For SEM pictures and immunoblots detecting ß-lactamase and TET8, apoplastic fluids were additionally ultracentrifuged for 1 hour at 100,000× *g*. The pellet was resuspended in 100 µL particle-free 1 mM EDTA.

### EV quantification, size, charge measurements, and proteinase K treatment

EVs were quantified and had their size and charge measured by NTA using ZetaView BASIC PMX-120 (Particle Metrix, Germany) at room temperature. To detect EVs, we used the manufacturer’s default settings for liposomes. Particle quantification and size measurements were performed by scanning 11 cell positions each and capturing 30 frames per position with the following settings: focus: autofocus; camera sensitivity for all samples: 85; shutter: 100; scattering intensity: detected automatically. After capture, the videos were analyzed by the built-in ZetaView Software 8.05.11 (ZNTA) with the following specific analysis parameters: maximum area: 1,000; minimum area: 5; minimum brightness: 25; trace length: 15 ms; hardware: embedded laser: 40 mW at 488 nm; camera: CMOS. For particle charge measurements, the same settings were used except minimum brightness: 30. Statistical analysis was performed using either one-way analysis of variance (ANOVA) with Tukey post hoc test or Welsch’s ANOVA with Dunnett’s T3 multiple comparisons post hoc test.

All samples were diluted in particle-free 1 mM EDTA buffer and checked with NTA. Unconditioned KB medium contained up to 1.4 × 10^9^ particles (Fig. S2A). *Pto* DC3000 cultures contained increasing particle numbers with cultivation time: ≈2.8 × 10^9^ particles at 1.5–2 × 10^9^ cfu/mL (50% of influence); ≈3.7 × 10^9^ particles at 2.25–2.75 × 10^9^ cfu/mL (37% of influence), ≈7 × 10^9^ particles at 3.75–4.5 cfu/mL (20% of influence), and ≈1.1 × 10^10^ particles at 5–5.5 × 10^9^ cfu/mL (13% of influence) (Fig. S2A). We, therefore, focused our measurements on samples collected from OD_600_ >7.5, which shows lower than 20% influence of particles from the medium. We calculated the colony-forming units per milliliter from measured OD_600_ values (71, 72).

For proteinase K treatment, EVs were incubated with 10 µg/mL proteinase K (NEB, P8107S) or mock treated for 30 minutes at 37°C before boiling in Lämmli buffer at 95°C for 5 minutes.

### Propidium iodide staining

The viability assay was done with some modifications according to reference (73). In brief, *Pto* DC3000 cultures were grown until OD_600_ = 1–2 and OD_600_ = 3–4. Propidium iodide (PI) (Sigma-Aldrich) was added to a final concentration of 20 µM. After 10 minutes incubation time, 5 µL of the stained cultures was transferred to a microscopy slide, and pictures were obtained with a Leica 3D Assay THUNDER Imager (Leica, Wetzlar) using an HC PL Fluotar L 40×/0.60 dry objective. PI was excited at 642 nm, and the emission range was 100%. As a negative control, bacteria were boiled in a microwave for several minutes before PI staining. Two technical replicates were performed for OD_600_ = 1–2 and OD_600_ = 3–4, respectively.

### Scanning electron microscopy

Planktonic-grown bacteria at OD_600_ = 3–4 (1.5–2 × 10^9^ cfu/mL), gradient-collected EVs (0.5–1.5 × 10^10^ particles), and apoplastic fluids passed through 0.2 µm filters were used for SEM. The cells were chemically fixed using 2.5% glutaraldehyde in 50 mM cacodylate buffer (pH 7.0) containing 2 mM MgCl_2_. Then, the cells were applied to a glass slide, covered with a cover slip, and plunged frozen in liquid nitrogen. After this, the cover slip was removed, and the cells were placed in a fixation buffer again. After washing four times with buffer, post-fixation was carried out with 1% OsO_4_ for 15 minutes. Two additional washing steps with buffer were followed by three times washing with double distilled water. The samples were dehydrated in a graded acetone series, critical point dried, and mounted on an aluminium stub. To enhance conductivity, the samples were sputter coated with platinum. Microscopy was carried out using a Zeiss Auriga Crossbeam workstation at 2 kV (Zeiss, Oberkochen, Germany). The vesicle size was manually measured across five randomly selected SEM micrographs using Fiji software (74).

### Transmission electron microscopy

Planktonic-grown *Pto* DC3000 at OD_600_ = 3–4 (1.5–2 × 10^9^ cfu/mL) was used for ultrathin sectioning and subsequent TEM. The cells were concentrated by centrifugation, and the cells were high-pressure frozen using a Leica HPM100 (Leica Microsystems, Wetzlar, Germany). This was followed by freeze substitution with 0.2% osmium tetroxide, 0.1% uranyl acetate, and 9.3% water in water-free acetone in a Leica AFS 2 (Leica Microsystems, Wetzlar, Germany) as described previously (75). After embedding in Epon 812 substitute resin (Fluka Chemie AG, Buchs Switzerland), the cells were ultrathin sectioned (50–100 nm thickness) and post-stained for 1 minute with lead citrate. TEM of ultrathin sections was carried out with a JEOL F200 cryo-S(TEM), which was operated at 200 kV and at room temperature in the TEM mode. Images were acquired using a bottom-mounted XAROSA 20 mega-pixel CMOS camera (EMSIS, Münster, Germany).

For immuno-negative staining, freshly purified EVs from *Pto* DC3000 were used. Herein, 15 µL sample was applied to 175 mesh nickel grids, which had been covered with collodium plastic foil and coated with carbon in advance. After incubation for 5 minutes, the grids were blocked for 25 and 30 minutes with 0.1% BSA (bovine serum albumin) in 1× PBS. After this, the grids were incubated with the primary antibody (rabbit anti-*P*. *aeruginosa* ampC polyclonal antibody, dilution 1:1,000 or 1:5,000 in 1× PBS) for 30 minutes. This was followed by washing six times for 5 minutes with 0.1% BSA in 1× PBS before adding the secondary antibody (goat-anti-rabbit, coupled to 10 nm colloidal gold, dilution: 1:20) for 30 minutes. After this, the grids were washed 2 × 5 minutes with 0.1% BSA in 1× PBS, 2 × 5 minutes with 1× PBS, and 2 × 5 minutes with sterile water. After blocking with a filter paper, the samples were negatively stained with 1% uranyl acetate for 2 minutes, blotted again on a filter paper, and air dried.

As it is expected that the epitope for immunodetection is on the inside of the EVs, we carried out a heat treatment in parallel to break the EVs open. For this, 15 µL of the sample was applied to 400 mesh carbon-coated copper grids and incubated for 20 minutes at 120°C. From this point on, the treatment of the grids was identical to the protocol above.

The immuno-negative stained samples were investigated using a Zeiss EM912 (Zeiss, Oberkochen, Germany) at 80 kV acceleration voltage. Images were acquired using a 2k × 2k slow-speed CCD camera (TRS Tröndle Restlichverstärker-Systeme, Moorenweis, Germany).

### *Pto* DC3000 infection assay

Overnight plate-grown *Pto* DC3000 cells were resuspended in 10  mM MgCl_2_ and diluted to OD_600_ = 0.0006. Using a needle-less syringe, the bacterial suspension was infiltrated into mature leaves of 5–6-week-old plants, three leaves per plant. For pretreatments, gradient-collected EVs from planktonic *Pto* DC3000 (concentration ≈1.10^10^) and 0.02 mM EDTA as a negative control and 100 nM flg22 (EZbiolabs) as a positive control were syringe infiltrated into leaves 24 hours prior to *Pto* DC3000 inoculation. Discs of the infected leaves (one disc per leaf, 0.6 cm diameter) were excised at 1, 2, or 3 dpi. The three leaf discs from each plant were pooled and ground in 1 mL 10 mM MgCl_2_. Serial dilutions were plated on LB medium with rifampicin (50 µg/mL), and bacterial colonies were counted 1 day after incubation at 28°C. Statistical analysis was performed using a two-tailed Welsch’s t-test.

### Histochemical GUS staining

The histochemical GUS assay was performed with 11-day-old seedlings. Seedlings were treated with gradient-collected *Pto* DC3000 EVs (concentration ≈1.10^10^), 100 nM flg22 (EZbiolabs), or as a control with 0.02 mM EDTA for 18 hours. Treated seedlings were immersed in X-Gluc buffer [2 mM X-Gluc (Biosynth), 50 mM NaPO_4_, pH 7, 0.5% (vol/vol) Triton-X100, 0.5 mM K-ferricyanide] for 16 hours at 37°C. Chlorophyll was removed by repeated washing in 80% (vol/vol) ethanol. Observations were made on a WHX 6000 digital microscopy (76). The intensity of the GUS signals was quantified using ImageJ software as described in reference (77).

### Fluorimetric GUS assay

For fluorimetric GUS assays, 11–12-day-old seedlings were treated with gradient-collected *Pto* DC3000 EVs (concentration ≈1.10^10^) or with 100 nM flg22 (EZbiolabs) or as a control with 0.02 mM EDTA for 18 hours. Treated seedlings were frozen in liquid nitrogen in 2 mL conical tubes containing two clean sterile glass beads and liquid nitrogen. The frozen samples were dry homogenized using a Retch mixer mill (Retch). Homogenized samples were kept on ice and cold (4°C). For total protein extraction, GUS extraction buffer was added as described (78) [50 mM sodium phosphate (pH 7); 10 mM 2-mercaptoethanol; 10mM Na_2_EDTA; 0.1% Triton X-100; 0.1% sodium lauryl-sarcosine and PPIC (plant protease inhibitor cocktail)]. GUS activities were measured fluorimetrically in reaction buffer (see below) using methylumbelliferyl-β-D-glucuronic acid dihydrate (MUG) (Biosynth) as a substrate. Reaction buffer was the same solution as extraction buffer with one modification: PPIC was replaced by 1 mM MUG. The fluorescence was measured using TECAN fluorimeter at excitation 360 nm and emission 465 nm. The enzymatic activity of the sample was calculated to protein concentration measured by Bradford protein assay. The absorbance was measured using TECAN spectrometer absorbance at 595 nm. Statistical analysis was performed using one-way ANOVA with Tukey post hoc test.

### RNA extraction and RT-qPCR analysis

Gene transcription analysis was performed with 12-day-old seedlings. The seedlings were treated with gradient-enriched EVs (concentration 1.10^10^) and 0.02 mM EDTA as control for 3 hours, frozen in liquid nitrogen, and ground with 2.5-mm-diameter silica beads using a homogenizer (Retch, Germany). Total RNA was isolated using a TRIzol reagent (Invitrogen, USA) according to the manufacturer’s protocol. The extracted RNA was treated with a DNA-free kit (Ambion, USA). Subsequently, 1 µg of RNA was converted into cDNA with M-MLV RNase H—Point Mutant reverse transcriptase (Promega Corp., USA) and an anchored oligo dT21 primer (Metabion, Germany). Gene transcription was quantified by qPCR using a LightCycler 480 SYBR Green I Master kit and LightCycler 480 (Roche, Switzerland). The PCR conditions were 95°C for 10 minutes followed by 45 cycles of 95°C for 10 seconds, 55°C for 20 seconds, and 72°C for 20 seconds. Melting curve analyses were then carried out. Relative transcription was normalized to the housekeeping gene AtTIP41 (79). Primers were designed using PerlPrimer v1.1.21 (80). The primers used are AtFRK1_FP, GCCAACGGAGACATTAGAG and AtFRK1_RP, CCATAACGACCTGACTCATC. Statistical analysis was performed using one-way ANOVA with Tukey post hoc test.

### Seedling growth analysis

Four-day-old seedlings were transferred from MS solid media into the liquid MS media in transparent 96-well microplates. Each well contained 100 µL of media either containing 0.02 mM EDTA as a control or gradient-collected *Pto* DC3000 EVs (concentration ≈1.10^10^) or with 100 nM flg22 (EZbiolabs) as a positive control. After 8 days, the treated seedlings were dried using a paper towel and then the fresh weight was measured. Based on the weight of each seedling, relative seedling growth (%) to control seedlings was calculated. Statistical analysis was performed using Welsch’s ANOVA with Dunnett’s T3 multiple comparisons post hoc test two-tailed Student t-test.

### ROS measurements

ROS production was determined using the luminol-based assay as previously described (81). Briefly, leaves of 5–6-week-old *A. thaliana* plants were infiltrated with gradient-collected EVs (concentration ≈1.10^10^) or particles isolated from KB. After 2 hours, discs were excised from the infiltrated leaves and 24 hours incubated in ddH2O at 22°C. Then, the leaf discs were treated with water as mock and with 100 nM flg22 or 100 nM elf18 (EZbiolabs) to induce the production of ROS. The total photon count was collected for 45 min using a TECAN luminometer. Statistical analysis was performed using a two-tailed Student t-test.

### Proteomics

We isolated proteins in parallel from *Pto* DC3000 WC lysates and OM and EVs as described previously (51, 82) and above, respectively. WC, OM, and EVs were isolated from *Pto* DC3000 liquid cultures (OD_600_ = 3–4 (1.5–2 × 10^9^ cfu/mL). The cells were pelleted via centrifugation (12,000 × *g* for 10 minutes).

Briefly, for WC, the pellet was resuspended in 1 mL of 20 mM Tris-HCl (pH 8.0), frozen in liquid nitrogen, three times thawing-freezing, and three times sonicated for 10 minutes at 4°C. The samples were centrifuged at 6,000 × *g* for 10 minutes at 4°C, and supernatants were collected and frozen in liquid nitrogen.

For OM preparations, the pellet was resuspended in 1 mL 20 mM Tris-HCl (pH 8.0), sucrose (20%), followed by adding 5 µL lysozyme (15 mg/mL) and 10 µL 0.5 M EDTA, incubation for 40 minutes on ice, and adding 20 µL 0.5 M MgCl_2_. After centrifugation at 9,500 × *g* for 20 minutes at 4°C, the pellet was resuspended in 1 mL ice-cold 10 mM Tris-HCl (pH 8.0) followed by sonication three times for 10 minutes on ice. The samples were then centrifuged at 8,000 × *g* for 5 minutes at 4°C, washed with cold 10 mM Tris-HCl (pH 8.0), resuspended in cold, sterile MilliQ water followed by three times freezing thawing in liquid nitrogen, incubation for 20 minutes at 25°C, and adding the sarcosyl to final concentration 0.5%. The samples were then centrifuged at 40,000 × *g* for 90 minutes at 4°C, the pellet was resuspended in ice-cold 10 mM Tris-HCl (pH 8.0), and frozen in liquid nitrogen.

Gradient-collected EVs were isolated from the bacteria cultures as described above (Fig. S1C). The protein concentration in the samples was measured using Bio-Rad Protein Assay which is based on Bradford method (83).

For proteomics, the samples were denatured by addition of 1× SDS loading buffer. In-gel trypsin digestion was performed according to standard procedures (84). Briefly, 2 µg of EV and OM samples and 20 µg of WC samples were loaded on a NuPAGE 4%–12% Bis-Tris Protein gels (Thermofisher Scientific, USA), and the gels were run for 3 minutes only. Subsequently, the still not size-separated single protein band per sample was cut, reduced (50 mM DTT), alkylated (55 mm CAA, chloroacetamide), and digested overnight with trypsin (trypsin-gold, Promega).

### LC-MS/MS data acquisition

Peptides generated by in-gel trypsin digestion were dried in a vacuum concentrator and dissolved in 0.1% formic acid (FA). LC-MS/MS measurements were performed on a Fusion Lumos Tribrid mass spectrometer (Thermo Fisher Scientific) equipped with an Ultimate 3000 RSLCnano system. Peptides were delivered to a trap column (ReproSil-pur C18-AQ, 5 µm, Dr Maisch, 20 mm × 75 μm, self-packed) at a flow rate of 5 µL/minute in 100% solvent A (0.1% FA in HPLC grade water). After 10minutes of loading, peptides were transferred to an analytical column (ReproSil Gold C18-AQ, 3µm, Dr Maisch, 400mm × 75 μm, self-packed) and separated using a 50-minute gradient from 4% to 32% of solvent B [0.1% FA in acetonitrile and 5% (vol/vol) DMSO] at 300nL/minute flow rate. Both nanoLC solvents contained 5% (vol/vol) DMSO.

The Fusion Lumos Tribrid mass spectrometer was operated in data-dependent acquisition and positive ionization mode. MS1 spectra (360–1,300 m/z) were recorded at a resolution of 60,000 using an automatic gain control (AGC) target value of 4e^5^ and maximum injection time (maxIT) of 50 ms. After peptide fragmentation using higher-energy collision-induced dissociation, MS2 spectra of up to 20 precursor peptides were acquired at a resolution of 15,000 with an AGC target value of 5e^4^ and maxIT of 22 ms. The precursor isolation window width was set to 1.3 m/z and normalized collision energy to 30%. Dynamic exclusion was enabled with 20-second exclusion time (mass tolerance ±10 ppm).

### Computational analysis of proteomes

LFQ values were used in the statistical analysis of proteome data. To select EV-enriched proteins, Welch t-test was used to compare protein intensities between EV and WC samples. The resulting *P*-values were corrected using the Benjamini–Hochberg (BH) method to control the FDR. The proteins with FDR <0.05 and with the intensity in EV at least twice higher than in WC were selected as EV-enriched proteins (*n* = 207). In addition, we selected proteins that were exclusively identified in at least three (out of four) replicates of EV (*n* = 162). A complete list of EV-enriched proteins is given in Table S1. The functional enrichment analysis of the EV proteins was performed using the DAVID functional annotation tool (36, 37). Cluster maps were generated using the SEABORN python library (https://seaborn.pydata.org/), with small cosmetic changes based on its documentation. Gene clusters were generated by SEABORN default method of hierarchical clustering. These gene clusters were also subjected to functional enrichment analysis using the DAVID functional annotation tool (36, 37).

### Database searches

Peptide identification and quantification were performed using MaxQuant (version 1.6.3.4) with its built-in search engine Andromeda (85, 86). MS2 spectra were searched against a *Pto* protein database (UP000002515, downloaded from Uniprot 04.05.2020) supplemented with common contaminants (built-in option in MaxQuant). For all MaxQuant searches, default parameters were employed. Those included carbamidomethylation of cysteine as a fixed modification and oxidation of methionine and N-terminal protein acetylation as variable modifications. Trypsin/P was specified as a proteolytic enzyme. Precursor tolerance was set to 4.5 ppm, and fragment ion tolerance to 20 ppm. Results were adjusted to 1% FDR on peptide spectrum match and protein level, employing a target-decoy approach using reversed protein sequences. LFQ algorithm was enabled. The minimal peptide length was defined as seven amino acids, and the “match-between-run” function was not enabled. Each sample type (EV, OM, WC) was analyzed in biological quadruplicates (Table S1).

We used available localization prediction data from the *Pseudomonas* genome database (pseudomonas.com) (87). Predicted protein localizations are presented as stacked bar charts (made in MS Excel) as a percentage of the total number of proteins in the analyzed sample. We used the available software DAVID bioinformatic resource 6.8 (https://david.ncifcrf.gov/) for GO term and KEGG (Kyoto Encyclopedia of Genes and Genomes) pathway analysis, and the adjusted *P*-value cutoff was set to 0.05 (36, 37). We compared the EV-enriched proteins from *Pto* DC3000 with EV proteomes from planktonic-grown *P. aeruginosa* PAO1 (51, 52, 82). We focused on the proteins that were identified in OMVs from *P. aeruginosa* PAO1 across all three studies and identified their gene orthologs in *Pto* DC3000 using the *Pseudomonas* genome database (pseudomonas.com) (87). This set of proteins was compared with the *Pto* DC3000 EV-enriched proteins to predict EV biomarkers. The EV-enriched proteins were also compared with available *in planta Pto* DC3000 transcriptome and proteome datasets (11, 33).

### Immunoblot analysis

Immunoblot analysis was performed according to Sambrook and Russel (1989) (88). And 10% SDS-PAGE gels were blotted onto PVDF Immobilon-P membranes (Millipore). *Pto* DC3000 OprF was detected using 1:2,000 diluted rabbit polyclonal antibody against OprF from *P. aeruginosa* (Cusabio Biotech Co.; CSB-PA318417LA01EZX); flagellin was detected using 1:300 diluted antibody (68); β-lactamase was detected using 1:2,000 diluted rabbit polyclonal antibody against ampC from *P. aeruginosa* (Cusabio Biotech Co.; CSB-PA326492HA01EZX); and TET8 was detected using 1:500 diluted rabbit polyclonal antibody against TET8 from *Arabidopsis* (PhytoAB, PHY1490S). As secondary antibody, we used a 1:50,000 dilution of the anti-rabbit IgG-peroxidase polyclonal antibody (Sigma-Aldrich, A0545) and 1:5,000 dilution of IRDye 800CW Goat anti-Rabbit IgG Secondary Antibody (LI-COR Biosciences, 926–32211). Signal detection was done using SuperSignal West FemtoMaximum Sensitivity Substrate (Pierce, Thermo Scientific), according to the manufacturer’s instructions, and the images were captured using Vilber Lourmat Peqlab FUSION SL Gel Chemiluminescence Documentation System. For detection of the IRDye 800CW Goat anti-Rabbit IgG Secondary Antibody, we used the Odyssey CLx Near-Infrared Fluorescence Imaging System Odyssey Clx (LI-COR, Biosciences).

### Coomassie brilliant blue and silver staining

Proteins were separated on 10% SDS-PAGE gels using Hoefer’s vertical electrophoresis system (SE250, Hoefer). The gels were subsequently either incubated with Coomassie brilliant blue G-250 staining buffer at room temperature, or the silver staining was performed using ROTI Black P kit (L533, Carl Roth) following the protocol provided by the manufacturer.

### Statistical analysis

Student *t-*test, Welsch’s t-test, one-way ANOVA followed by Tukey multiple comparisons test, and Welsch’s ANOVA with Dunnett’s T3 multiple comparisons post hoc test were performed using GraphPad Prism version 8.3 for Windows, GraphPad Software, San Diego, CA, USA, www.graphpad.com.

## Data Availability

The mass spectrometry proteomics data have been deposited to the ProteomeXchange Consortium via the PRIDE (89) partner repository with the dataset identifier PXD023971.

## SUPPLEMENTAL MATERIAL

Supplemental legends and Figure S1 mbio.03589-22-s0001.pdf Supplemental legends; observation and isolation of *Pto* DC3000 EVs.

Figure S2 mbio.03589-22-s0002.pdf Characteristics of *Pto* DC3000 EV isolation.

Figure S3 mbio.03589-22-s0003.pdf Characteristics of the proteomic analysis.

Figure S4 mbio.03589-22-s0004.pdf Immunogenicity of *Pto* DC3000 EVs.

Figure S5 mbio.03589-22-s0005.pdf Isolation and observation of vesicles in apoplastic fluids.

Figure S6 mbio.03589-22-s0006.pdf Biophysical parameters of particles in apoplastic fluids from *A. thaliana* plants infected with *Pto* DC3000.

Figure S7 mbio.03589-22-s0007.pdf Selecting candidate EV biomarkers.

Figure S8 mbio.03589-22-s0008.pdf Biophysical parameters of *Pto* DC3000 EVs across fractions from gradient enrichment.

Table S1 mbio.03589-22-s0009.xlsx Proteomics data.

Table S2 mbio.03589-22-s0010.xlsx GO analysis.

ASM does not own the copyrights to Supplemental Material that may be linked to, or accessed through, an article. The authors have granted ASM a non-exclusive, world-wide license to publish the Supplemental Material files. Please contact the corresponding author directly for reuse.

## References

[1] Büttner D , Bonas U . 2010. Regulation and secretion of Xanthomonas virulence factors. FEMS Microbiol Rev 34:107–133. doi:10.1111/j.1574-6976.2009.00192.x 19925633

[2] Mansfield J , Genin S , Magori S , Citovsky V , Sriariyanum M , Ronald P , Dow M , Verdier V , Beer SV , Machado MA , Toth I , Salmond G , Foster GD . 2012. Top 10 plant pathogenic bacteria in molecular plant pathology. Mol Plant Pathol 13:614–629. doi:10.1111/j.1364-3703.2012.00804.x 22672649 PMC6638704

[3] Wilson M , Campbell HL , Ji P , Jones JB , Cuppels DA . 2002. Biological control of bacterial speck of tomato under field conditions at several locations in North America. Phytopathology 92:1284–1292. doi:10.1094/PHYTO.2002.92.12.1284 18943882

[4] Melotto M , Underwood W , Koczan J , Nomura K , He SY . 2006. Plant stomata function in innate immunity against bacterial invasion. Cell 126:969–980. doi:10.1016/j.cell.2006.06.054 16959575

[5] Xin XF , He SY . 2013. Pseudomonas syringae pv. tomato DC3000: a model pathogen for probing disease susceptibility and hormone signaling in plants. Annu Rev Phytopathol 51:473–498. doi:10.1146/annurev-phyto-082712-102321 23725467

[6] Xin XF , Kvitko B , He SY . 2018. Pseudomonas syringae: what it takes to be a pathogen. Nat Rev Microbiol 16:316–328. doi:10.1038/nrmicro.2018.17 29479077 PMC5972017

[7] Yuan M , Jiang Z , Bi G , Nomura K , Liu M , Wang Y , Cai B , Zhou JM , He SY , Xin XF . 2021. Pattern-recognition receptors are required for NLR-mediated plant immunity. Nature 592:105–109. doi:10.1038/s41586-021-03316-6 33692546 PMC8016741

[8] Couto D , Zipfel C . 2016. Regulation of pattern recognition receptor signalling in plants. Nat Rev Immunol 16:537–552. doi:10.1038/nri.2016.77 27477127

[9] Dodds PN , Rathjen JP . 2010. Plant immunity: towards an integrated view of plant-pathogen interactions. Nat Rev Genet 11:539–548. doi:10.1038/nrg2812 20585331

[10] Kvitko BH , Park DH , Velásquez AC , Wei C-F , Russell AB , Martin GB , Schneider DJ , Collmer A . 2009. Deletions in the repertoire of Pseudomonas syringae pv. tomato DC3000 type III secretion effector genes reveal functional overlap among effectors. PLoS Pathog 5:e1000388. doi:10.1371/journal.ppat.1000388 19381254 PMC2663052

[11] Nobori T , Wang Y , Wu J , Stolze SC , Tsuda Y , Finkemeier I , Nakagami H , Tsuda K . 2020. Multidimensional gene regulatory landscape of a bacterial pathogen in plants. Nat Plants 6:1064. doi:10.1038/s41477-020-0746-8 32541952

[12] Nobori T , Wang Y , Wu J , Stolze SC , Tsuda Y , Finkemeier I , Nakagami H , Tsuda K . 2019 In planta bacterial multi-omics analysis illuminates regulatory principles underlying plant-pathogen interactions. Plant Biol. doi:10.1101/822932

[13] Nomura K , Debroy S , Lee YH , Pumplin N , Jones J , He SY . 2006. A bacterial virulence protein suppresses host innate immunity to cause plant disease. Science 313:220–223. doi:10.1126/science.1129523 16840699

[14] Schwechheimer C , Kuehn MJ . 2015. Outer-Membrane vesicles from Gram-negative bacteria: biogenesis and functions. Nat Rev Microbiol 13:605–619. doi:10.1038/nrmicro3525 26373371 PMC5308417

[15] Bielska E , Birch PRJ , Buck AH , Abreu-Goodger C , Innes RW , Jin H , Pfaffl MW , Robatzek S , Regev-Rudzki N , Tisserant C , Wang S , Weiberg A . 2019. Highlights of the mini-symposium on extracellular vesicles in inter-organismal communication, held in Munich, Germany, August 2018. J Extracell Vesicles 8:1590116. doi:10.1080/20013078.2019.1590116 30911363 PMC6427632

[16] Rybak K , Robatzek S . 2019. Functions of extracellular vesicles in immunity and virulence. Plant Physiol 179:1236–1247. doi:10.1104/pp.18.01557 30705070 PMC6446742

[17] Raposo G , Stoorvogel W . 2013. Extracellular vesicles: exosomes, microvesicles, and friends. J Cell Biol 200:373–383. doi:10.1083/jcb.201211138 23420871 PMC3575529

[18] Roier S , Zingl FG , Cakar F , Durakovic S , Kohl P , Eichmann TO , Klug L , Gadermaier B , Weinzerl K , Prassl R , Lass A , Daum G , Reidl J , Feldman MF , Schild S . 2016. A novel mechanism for the biogenesis of outer membrane vesicles in gram-negative bacteria. Nat Commun 7:10515. doi:10.1038/ncomms10515 26806181 PMC4737802

[19] Pérez-Cruz C , Delgado L , López-Iglesias C , Mercade E . 2015. Outer-inner membrane vesicles naturally secreted by gram-negative pathogenic bacteria. PLoS One 10:e0116896. doi:10.1371/journal.pone.0116896 25581302 PMC4291224

[20] Toyofuku M , Nomura N , Eberl L . 2019. Types and origins of bacterial membrane vesicles. Nat Rev Microbiol 17:13–24. doi:10.1038/s41579-018-0112-2 30397270

[21] Roszkowiak J , Jajor P , Guła G , Gubernator J , Żak A , Drulis-Kawa Z , Augustyniak D . 2019. Interspecies outer membrane Vesicles (Omvs) modulate the sensitivity of pathogenic bacteria and pathogenic yeasts to Cationic peptides and serum complement. Int J Mol Sci 20:5577. doi:10.3390/ijms20225577 31717311 PMC6888958

[22] Kaparakis-Liaskos M , Ferrero RL . 2015. Immune modulation by bacterial outer membrane vesicles. Nat Rev Immunol 15:375–387. doi:10.1038/nri3837 25976515

[23] McMillan HM , Kuehn MJ . 2021. The extracellular vesicle generation paradox: a bacterial point of view. EMBO J 40:e108174. doi:10.15252/embj.2021108174 34636061 PMC8561641

[24] McMillan HM , Rogers N , Wadle A , Hsu-Kim H , Wiesner MR , Kuehn MJ , Hendren CO . 2021. Microbial vesicle-mediated communication: convergence to understand interactions within and between domains of life. Environ Sci Process Impacts 23:664–677. doi:10.1039/d1em00022e 33899070

[25] Bahar O , Mordukhovich G , Luu DD , Schwessinger B , Daudi A , Jehle AK , Felix G , Ronald PC . 2016. Bacterial outer membrane vesicles induce plant immune responses. Mol Plant Microbe Interact 29:374–384. doi:10.1094/MPMI-12-15-0270-R 26926999

[26] Feitosa-Junior OR , Stefanello E , Zaini PA , Nascimento R , Pierry PM , Dandekar AM , Lindow SE , da Silva AM . 2019. Proteomic and metabolomic analyses of Xylella fastidiosa OMV-enriched fractions reveal association with virulence factors and signaling molecules of the DSF family. Phytopathology 109:1344–1353. doi:10.1094/PHYTO-03-19-0083-R 30973310

[27] Chowdhury C , Jagannadham MV . 2013. Virulence factors are released in association with outer membrane vesicles of Pseudomonas syringae pv. tomato T1 during normal growth. Biochim Biophys Acta 1834:231–239. doi:10.1016/j.bbapap.2012.09.015 23043909

[28] Sidhu VK , Vorhölter F-J , Niehaus K , Watt SA . 2008. Analysis of outer membrane vesicle associated proteins isolated from the plant pathogenic bacterium Xanthomonas campestris pv. campestris. BMC Microbiol 8:87. doi:10.1186/1471-2180-8-87 18518965 PMC2438364

[29] Mitre LK , Teixeira-Silva NS , Rybak K , Magalhães DM , de Souza-Neto RR , Robatzek S , Zipfel C , de Souza AA . 2021. The Arabidopsis immune receptor EFR increases resistance to the bacterial pathogens Xanthomonas and Xylella in transgenic sweet orange. Plant Biotechnol J 19:1294–1296. doi:10.1111/pbi.13629 33991397 PMC8313127

[30] McMillan HM , Zebell SG , Ristaino JB , Dong X , Kuehn MJ . 2021. Protective plant immune responses are elicited by bacterial outer membrane vesicles. Cell Rep 34:108645. doi:10.1016/j.celrep.2020.108645 33472073 PMC8158063

[31] Klimentová J , Stulík J . 2015. Methods of isolation and purification of outer membrane vesicles from gram-negative bacteria. Microbiol Res 170:1–9. doi:10.1016/j.micres.2014.09.006 25458555

[32] Bachurski D , Schuldner M , Nguyen P-H , Malz A , Reiners KS , Grenzi PC , Babatz F , Schauss AC , Hansen HP , Hallek M , Pogge von Strandmann E . 2019. Extracellular vesicle measurements with nanoparticle tracking analysis - an accuracy and repeatability comparison between Nanosight NS300 and Zetaview. J Extracell Vesicles 8:1596016. doi:10.1080/20013078.2019.1596016 30988894 PMC6450530

[33] Nobori T , Velásquez AC , Wu J , Kvitko BH , Kremer JM , Wang Y , He SY , Tsuda K . 2018. Transcriptome landscape of a bacterial pathogen under plant immunity. Proc Natl Acad Sci U S A 115:E3055–E3064. doi:10.1073/pnas.1800529115 29531038 PMC5879711

[34] Ashburner M , Ball CA , Blake JA , Botstein D , Butler H , Cherry JM , Davis AP , Dolinski K , Dwight SS , Eppig JT , Harris MA , Hill DP , Issel-Tarver L , Kasarskis A , Lewis S , Matese JC , Richardson JE , Ringwald M , Rubin GM , Sherlock G . 2000. Gene ontology: tool for the unification of biology.The Gene Ontology Consortium. Nat Genet 25:25–29. doi:10.1038/75556 10802651 PMC3037419

[35] The Gene Ontology Consortium . 2019. The gene ontology resource: 20 years and still going strong. Nucleic Acids Res 47:D330–D338. doi:10.1093/nar/gky1055 30395331 PMC6323945

[36] Huang DW , Sherman BT , Lempicki RA . 2009. Systematic and integrative analysis of large gene Lists using DAVID bioinformatics resources. Nat Protoc 4:44–57. doi:10.1038/nprot.2008.211 19131956

[37] Huang DW , Sherman BT , Lempicki RA . 2009. Bioinformatics enrichment tools: paths toward the comprehensive functional analysis of large gene Lists. Nucleic Acids Res 37:1–13. doi:10.1093/nar/gkn923 19033363 PMC2615629

[38] Kulp A , Kuehn MJ . 2010. Biological functions and biogenesis of secreted bacterial outer membrane vesicles. Annu Rev Microbiol 64:163–184. doi:10.1146/annurev.micro.091208.073413 20825345 PMC3525469

[39] Kramer J , Özkaya Ö , Kümmerli R . 2020. Bacterial siderophores in community and host interactions. Nat Rev Microbiol 18:152–163. doi:10.1038/s41579-019-0284-4 31748738 PMC7116523

[40] Manabe T , Kato M , Ueno T , Kawasaki K . 2013. Flagella proteins contribute to the production of outer membrane vesicles from Escherichia coli W3110. Biochem Biophys Res Commun 441:151–156. doi:10.1016/j.bbrc.2013.10.022 24134841

[41] Bredow M , Sementchoukova I , Siegel K , Monaghan J . 2019. Pattern-triggered oxidative burst and Seedling growth inhibition assays in Arabidopsis Thaliana. J Vis Exp. doi:10.3791/59437 31180345

[42] Zipfel C , Robatzek S , Navarro L , Oakeley EJ , Jones JDG , Felix G , Boller T . 2004. Bacterial disease resistance in Arabidopsis through flagellin perception. Nature 428:764–767. doi:10.1038/nature02485 15085136

[43] Wang Y , Garrido-Oter R , Wu J , Winkelmüller TM , Agler M , Colby T , Nobori T , Kemen E , Tsuda K . 2019. Site-specific cleavage of bacterial Mucd by secreted proteases mediates Antibacterial resistance in Arabidopsis. Nat Commun 10:2853. doi:10.1038/s41467-019-10793-x 31253808 PMC6599210

[44] Vinatzer BA , Teitzel GM , Lee MW , Jelenska J , Hotton S , Fairfax K , Jenrette J , Greenberg JT . 2006. The type III effector repertoire of Pseudomonas syringae pv. syringae B728a and its role in survival and disease on host and non-host plants. Mol Microbiol 62:26–44. doi:10.1111/j.1365-2958.2006.05350.x 16942603

[45] Lovelace AH , Smith A , Kvitko BH . 2018. Pattern-triggered immunity alters the transcriptional regulation of virulence-associated genes and induces the sulfur starvation response in Pseudomonas syringae pv. tomato DC3000. Mol Plant Microbe Interact 31:750–765. doi:10.1094/MPMI-01-18-0008-R 29460676

[46] Schechter LM , Vencato M , Jordan KL , Schneider SE , Schneider DJ , Collmer A . 2006. Multiple approaches to a complete inventory of Pseudomonas syringae pv. tomato DC3000 type III secretion system effector proteins. Mol Plant Microbe Interact 19:1180–1192. doi:10.1094/MPMI-19-1180 17073301

[47] Block A , Alfano JR . 2011. Plant targets for Pseudomonas syringae type III effectors: virulence targets or guarded decoys? Curr Opin Microbiol 14:39–46. doi:10.1016/j.mib.2010.12.011 21227738 PMC3040236

[48] Tran TM , Chng CP , Pu X , Ma Z , Han X , Liu X , Yang L , Huang C , Miao Y . 2022. Potentiation of plant defense by bacterial outer membrane vesicles is mediated by membrane nanodomains. Plant Cell 34:395–417. doi:10.1093/plcell/koab276 34791473 PMC8846181

[49] Rutter BD , Innes RW . 2017. Extracellular vesicles isolated from the leaf apoplast carry stress-response proteins. Plant Physiol 173:728–741. doi:10.1104/pp.16.01253 27837092 PMC5210723

[50] Couto N , Schooling SR , Dutcher JR , Barber J . 2015. Proteome profiles of outer membrane vesicles and extracellular matrix of Pseudomonas aeruginosa biofilms . J. Proteome Res 14:4207–4222. doi:10.1021/acs.jproteome.5b00312 26303878

[51] Choi DS , Kim DK , Choi SJ , Lee J , Choi JP , Rho S , Park SH , Kim YK , Hwang D , Gho YS . 2011. Proteomic analysis of outer membrane vesicles derived from Pseudomonas aeruginosa . Proteomics 11:3424–3429. doi:10.1002/pmic.201000212 21751344

[52] Reales-Calderón JA , Corona F , Monteoliva L , Gil C , Martínez JL . 2015. Quantitative proteomics unravels that the post-transcriptional regulator CRC modulates the generation of vesicles and secreted virulence determinants of Pseudomonas aeruginosa. Data Brief 4:450–453. doi:10.1016/j.dib.2015.07.002 26306318 PMC4534582

[53] Toyofuku M , Roschitzki B , Riedel K , Eberl L . 2012. Identification of proteins associated with the Pseudomonas aeruginosa biofilm extracellular matrix. J Proteome Res 11:4906–4915. doi:10.1021/pr300395j 22909304

[54] Tamber SH , Robert EWH . 2004. The outer membranes of pseudomonads, . In Ramos J (ed), Pseudomonas. Springer. doi:10.1007/978-1-4419-9086-0

[55] Buzas EI . 2022. Opportunities and challenges in studying the extracellular vesicle corona. Nat Cell Biol 24:1322–1325. doi:10.1038/s41556-022-00983-z 36042293

[56] Devos S , Van Putte W , Vitse J , Van Driessche G , Stremersch S , Van Den Broek W , Raemdonck K , Braeckmans K , Stahlberg H , Kudryashev M , Savvides SN , Devreese B . 2017. Membrane vesicle secretion and prophage induction in multidrug-resistant Stenotrophomonas maltophilia in response to ciprofloxacin stress. Environ Microbiol 19:3930–3937. doi:10.1111/1462-2920.13793 28488744

[57] Orench-Rivera N , Kuehn MJ . 2016. Environmentally controlled bacterial vesicle-mediated export. Cell Microbiol 18:1525–1536. doi:10.1111/cmi.12676 27673272 PMC5308445

[58] Wan W-L , Fröhlich K , Pruitt RN , Nürnberger T , Zhang L . 2019. Plant cell surface immune receptor complex signaling. Curr Opin Plant Biol 50:18–28. doi:10.1016/j.pbi.2019.02.001 30878771

[59] Bauman SJ , Kuehn MJ . 2006. Purification of outer membrane vesicles from Pseudomonas aeruginosa and their activation of an IL-8 response. Microbes Infect 8:2400–2408. doi:10.1016/j.micinf.2006.05.001 16807039 PMC3525494

[60] Zingl FG , Kohl P , Cakar F , Leitner DR , Mitterer F , Bonnington KE , Rechberger GN , Kuehn MJ , Guan Z , Reidl J , Schild S . 2020. Outer membrane vesiculation facilitates surface exchange and in vivo adaptation of Vibrio cholerae. Cell Host Microbe 27:225–237. doi:10.1016/j.chom.2019.12.002 31901519 PMC7155939

[61] Devos S , Van Oudenhove L , Stremersch S , Van Putte W , De Rycke R , Van Driessche G , Vitse J , Raemdonck K , Devreese B . 2015. The effect of imipenem and diffusible signaling factors on the secretion of outer membrane vesicles and associated Ax21 proteins in Stenotrophomonas maltophilia. Front Microbiol 6:298. doi:10.3389/fmicb.2015.00298 25926824 PMC4396451

[62] Campos ML , de Souza CM , de Oliveira KBS , Dias SC , Franco OL . 2018. The role of antimicrobial peptides in plant immunity. J Exp Bot 69:4997–5011. doi:10.1093/jxb/ery294 30099553

[63] Vogel CM , Potthoff DB , Schäfer M , Barandun N , Vorholt JA . 2021. Protective role of the Arabidopsis leaf microbiota against a bacterial pathogen. Nat Microbiol 6:1537–1548. doi:10.1038/s41564-021-00997-7 34819644 PMC7612696

[64] Manning AJ , Kuehn MJ . 2011. Contribution of bacterial outer membrane Vesicles to innate bacterial defense. BMC Microbiol 11:258. doi:10.1186/1471-2180-11-258 22133164 PMC3248377

[65] Park J , Kim M , Shin B , Kang M , Yang J , Lee TK , Park W . 2021. A novel decoy strategy for polymyxin resistance in Acinetobacter Baumannii. Elife 10:e66988. doi:10.7554/eLife.66988 34180396 PMC8324293

[66] Aznar A , Dellagi A . 2015. New insights into the role of siderophores as triggers of plant immunity: what can we learn from animals? J Exp Bot 66:3001–3010. doi:10.1093/jxb/erv155 25934986

[67] Prados-Rosales R , Weinrick BC , Piqué DG , Jacobs WR , Casadevall A , Rodriguez GM . 2014. Role for Mycobacterium tuberculosis membrane vesicles in iron acquisition. J Bacteriol 196:1250–1256. doi:10.1128/JB.01090-13 24415729 PMC3957709

[68] Parys K , Colaianni NR , Lee H-S , Hohmann U , Edelbacher N , Trgovcevic A , Blahovska Z , Lee D , Mechtler A , Muhari-Portik Z , Madalinski M , Schandry N , Rodríguez-Arévalo I , Becker C , Sonnleitner E , Korte A , Bläsi U , Geldner N , Hothorn M , Jones CD , Dangl JL , Belkhadir Y . 2021. Signatures of antagonistic pleiotropy in a bacterial flagellin epitope. Cell Host Microbe 29:620–634. doi:10.1016/j.chom.2021.02.008 33713601

[69] Kunze G , Zipfel C , Robatzek S , Niehaus K , Boller T , Felix G . 2004. The N terminus of bacterial elongation factor Tu elicits innate immunity in Arabidopsis plants. Plant Cell 16:3496–3507. doi:10.1105/tpc.104.026765 15548740 PMC535888

[70] Zipfel C , Kunze G , Chinchilla D , Caniard A , Jones JDG , Boller T , Felix G . 2006. Perception of the bacterial PAMP EF-Tu by the receptor EFR restricts Agrobacterium-mediated transformation. Cell 125:749–760. doi:10.1016/j.cell.2006.03.037 16713565

[71] Jones AM , Wildermuth MC . 2011. The phytopathogen Pseudomonas syringae pv. tomato DC3000 has three high-affinity iron-scavenging systems functional under iron limitation conditions but dispensable for pathogenesis. J Bacteriol 193:2767–2775. doi:10.1128/JB.00069-10 21441525 PMC3133136

[72] Katagiri F , Thilmony R , He SY . 2002. The Arabidopsis thaliana-Pseudomonas Syringae interaction. Arabidopsis Book 1:e0039. doi:10.1199/tab.0039 22303207 PMC3243347

[73] Liang J , Klingl A , Lin Y-Y , Boul E , Thomas-Oates J , Marín M . 2019. A subcompatible rhizobium strain reveals infection duality in Lotus . J Exp Bot 70:1903–1913. doi:10.1093/jxb/erz057 30775775 PMC6436148

[74] Schindelin J , Arganda-Carreras I , Frise E , Kaynig V , Longair M , Pietzsch T , Preibisch S , Rueden C , Saalfeld S , Schmid B , Tinevez JY , White DJ , Hartenstein V , Eliceiri K , Tomancak P , Cardona A . 2012. Fiji: an open-source platform for biological-image analysis. Nat Methods 9:676–682. doi:10.1038/nmeth.2019 22743772 PMC3855844

[75] Flechsler J , Heimerl T , Pickl C , Rachel R , Stierhof YD , Klingl A . 2020. 2D and 3D immunogold localization on (epoxy) ultrathin sections with and without osmium tetroxide. Microsc Res Tech 83:691–705. doi:10.1002/jemt.23459 32057162

[76] Krcková Z , Kocourková D , Danek M , Brouzdová J , Pejchar P , Janda M , Pokotylo I , Ott PG , Valentová O , Martinec J . 2018. The Arabidopsis thaliana non-specific phospholipase C2 is involved in the response to Pseudomonas syringae attack. Ann Bot 121:297–310. doi:10.1093/aob/mcx160 29300825 PMC5808806

[77] Béziat C , Kleine-Vehn J , Feraru E . 2017. Histochemical staining of β-glucuronidase and its spatial quantification. Methods Mol Biol 1497:73–80. doi:10.1007/978-1-4939-6469-7_8 27864759

[78] Andriankaja A , Boisson-Dernier A , Frances L , Sauviac L , Jauneau A , Barker DG , de Carvalho-Niebel F . 2007. AP2-ERF transcription factors mediate Nod factor dependent Mt ENOD11 activation in root hairs via a novel cis-regulatory motif. Plant Cell 19:2866–2885. doi:10.1105/tpc.107.052944 17827349 PMC2048698

[79] Czechowski T , Stitt M , Altmann T , Udvardi MK , Scheible WR . 2005. Genome-Wide identification and testing of superior reference genes for transcript normalization in Arabidopsis. Plant Physiol 139:5–17. doi:10.1104/pp.105.063743 16166256 PMC1203353

[80] Marshall OJ . 2004. Perlprimer: Cross-platform, graphical Primer design for standard, Bisulphite and real-time PCR. Bioinformatics 20:2471–2472. doi:10.1093/bioinformatics/bth254 15073005

[81] Mersmann S , Bourdais G , Rietz S , Robatzek S . 2010. Ethylene signaling regulates accumulation of the FLS2 receptor and is required for the oxidative burst contributing to plant immunity. Plant Physiol 154:391–400. doi:10.1104/pp.110.154567 20592040 PMC2938167

[82] Park AJ , Murphy K , Krieger JR , Brewer D , Taylor P , Habash M , Khursigara CM . 2014. A temporal examination of the planktonic and biofilm proteome of whole cell Pseudomonas aeruginosa PAO1 using quantitative mass spectrometry. Molecular & Cellular Proteomics 13:1095–1105. doi:10.1074/mcp.M113.033985 24532839 PMC3977187

[83] Bradford MM . 1976. A rapid and sensitive method for the quantitation of microgram quantities of protein utilizing the principle of protein-dye binding. Anal Biochem 72:248–254. doi:10.1006/abio.1976.9999 942051

[84] Shevchenko A , Tomas H , Havlis J , Olsen JV , Mann M . 2006. In-gel digestion for mass spectrometric characterization of proteins and proteomes. Nat Protoc 1:2856–2860. doi:10.1038/nprot.2006.468 17406544

[85] Cox J , Neuhauser N , Michalski A , Scheltema RA , Olsen JV , Mann M . 2011. Andromeda: a peptide search engine integrated into the MaxQuant environment. J Proteome Res 10:1794–1805. doi:10.1021/pr101065j 21254760

[86] Tyanova S , Temu T , Cox J . 2016. The MaxQuant computational platform for mass spectrometry-based shotgun proteomics. Nat Protoc 11:2301–2319. doi:10.1038/nprot.2016.136 27809316

[87] Winsor GL , Griffiths EJ , Lo R , Dhillon BK , Shay JA , Brinkman FSL . 2016. Enhanced Annotations and features for comparing thousands of Pseudomonas Genomes in the Pseudomonas genome database. Nucleic Acids Res 44:D646–D653. doi:10.1093/nar/gkv1227 26578582 PMC4702867

[88] Sambrook J , Russell DW . 2001. Molecular cloning: A laboratory manual, . In Cold spring harbor laboratory, 3rd ed. Cold Spring Harbor, N.Y.

[89] Perez-Riverol Y , Csordas A , Bai J , Bernal-Llinares M , Hewapathirana S , Kundu DJ , Inuganti A , Griss J , Mayer G , Eisenacher M , Pérez E , Uszkoreit J , Pfeuffer J , Sachsenberg T , Yilmaz S , Tiwary S , Cox J , Audain E , Walzer M , Jarnuczak AF , Ternent T , Brazma A , Vizcaíno JA . 2019. The PRIDE database and related tools and resources in 2019: improving support for quantification data. Nucleic Acids Res 47:D442–D450. doi:10.1093/nar/gky1106 30395289 PMC6323896

