# Supplementary material for: Biophysical and proteomic analyses of Pseudomonas syringae pv. tomato DC3000 extracellular vesicles suggest adaptive functions during plant infection
Source: mBio. 2023 Jun 27;14(4):e03589-22. doi: 10.1128/mbio.03589-22 (PMC10470744; doi:10.1128/mbio.03589-22)
Supplement: Supplemental legends and Figure S1 — Supplemental legends; observation and isolation of Pto DC3000 EVs. [file mbio.03589-22-s0001.pdf]

Supplemental Information

Figure S1. Observation and isolation of *Pto* DC3000 EVs. A) The full-size SEM micrograph used in Fig. 1A of *Pto* DC3000 growth in planktonic culture (1.5-2x 10^9 cfu/mL). **B) B)** Size of EVs on *Pto* DC3000 surface of 17 bacteria was measured from nine independent SEM micrographs as shown in Fig. 1A (n = 254 EVs). SEM was done in three biological repeats with similar results. **C)** Schematic overview of EVs isolation from planktonic cultures for fluid sample and gradient-collected sample analysis. **D)** Size of EVs measured from SEM micrographs as shown in Fig. 1B (n = 153 EVs). The SEM was done in three biological repeats with similar results. The plotted boxes extend from 25th to 75th percentiles, whiskers go down to the minimal value and up to the maximal value, and the lines in the middle of the box represent the median. The dots in boxplots represent sizes of particular EVs.

Figure S2. Characteristics of *Pto* DC3000 EV isolation. A) Concentration of EVs from planktonic culture fluid samples and King's B (KB) medium (n=6-9) depending on bacterial colony forming units (cfu). **B)** Concentration of gradient-collected EVs depending on bacterial cfu. (n=5 for 0.5-1; n=20 for 1.5-2). **C, D)** Size and ζ-potential of EVs from planktonic bacterial cultures (enriched = gradient enriched 0.5-1x 10^9 (n=4) or 1.5-2x 10^9 (n=14) cfu/mL and fluid samples 3.8-5.5x 10^9 cfu/mL (n=9)). **E)** Propidium iodide (PI) staining of living and heat-killed *Pto* DC3000. Red colouring indicates dead bacteria stained by PI. Scale bar = 10 µm. **F)** Concentration of EVs from heat-killed *Pto* DC3000 (data for live bacteria are the same as in S2A). The plotted boxes extend from 25th to 75th percentiles, whiskers go down to the minimal value and up to the maximal value, and the lines in the middle of the box represent the median. The dots in boxplots represents values of particular biologically independent samples (n = 4-14).

Figure S3. Characteristics of the proteomic analysis. A) Bar plot shows the number of identified proteins in each replicate. The solid line indicates the cumulative protein IDs and dashed line shows the shared protein IDs. **B)** Boxplot shows a comparable distribution of protein intensities from each replicate. **C)** Predicted protein localization of identified in whole cell lysate (WC), outer membrane (OM), EVs, EV-enriched, EV unique-detected (the proteins identified only in EVs not in WC) and EV enriched – highly (protein which FDR < 0.005 and EV/WC > 20) in [%]. C = cytosolic; CM = cytoplasmic membrane; E = extracellular; OM = outer membrane; P = periplasm and U = unknown.

Figure S4. **Immunogenicity of *Pto* DC3000 EVs. A)** Representative pictures of *pFRK1*::GUS seedlings incubated without and with EVs (concentration ≈ 1.10^{10}) or with 100 nM flg22 for 18 h. The experiment was done in six biological repeats with similar results. **B)** Representative pictures of *pFRK1*::GUS seedlings incubated without and with purified EVs (concentration ≈ 1.10^{10}) Proteinase K (PK)-treated EVs, with 100 nM flg22, or particles purified from KB medium only for 24 h; using the procedure for EVs isolation from planktonic cultures and gradient enrichment (concentration ≈ 1.10^{10}) **C)** Representative pictures of *pFRK1*::GUS seedlings incubated without and with EVs (concentrations: ≈ 0.75, 1.25 and 2.5x 10^{10}) purified from *Pto* DC30000 wild type and Δ*fliC* cultures. Control = 0.2 µM EDTA; flg22 = 100 nM. The experiment was done in two biological repeats with similar results.

Figure S5. Isolation and observation of vesicles in apoplastic fluids. A, B) Representative SEM micrographs of apoplastic fluids collected from mock-treated leaves (A) or leaves infected with *Pto* DC3000 for 3 dpi (B). Observed structures are labelled and indicated with arrows. **C)** Size of EVs measured from 20 independent SEM micrographs as shown in Fig. S5A and S5B, and for comparison also including data from Fig. S1D; n = 72 for both AWF samples and n = 153 for "*in vitro*" samples.

Figure S6. Biophysical parameters of particles in apoplastic fluids from *A. thaliana* plants infected with *Pto* DC3000. A, D, G) Particle parameters over days post-infection (dpi). **B, E, H)** Particle parameters in response to inoculation with different *Pto* DC3000 densities. **C, F, I)** Particle parameters in response to inoculation with different *Pto* DC3000 and co-treatment with flg22. Each dot represents value of independent samples for size and ζ-potential it represents the median. For experiments were used 3 – 12 independent samples. **J)** and **K)** The profile of ζ-potential for each particle collected from apoplastic fluids of plants treated as indicated and gradient-collected EVs. Control = 0.2 µM EDTA; flg22 = 100 nM; n.t. = not treated; *Pto* DC3000 OD_{600} = 0.0006. Each treatment was 3 days long. The dots represent the mean across the ζ-potential values from independent samples: n = 8 (control); n = 10 (*Pto* DC3000); n = 6 (flg22); n = 4 (non-treatment). Experiments (A-K) were done at least in two biological repeats with similar results. The box in boxplots extends from 25th to 75th percentiles, whiskers go down to the minimal value and up to the maximal value, and the line in the middle of the box is plotted at the median. Different letters indicate significant difference (One-way ANOVA

with Tukey post hoc test; $p < 0.05$); no letters indicate no significant differences. The green colour is highlighting the particles from *Pto* DC3000 infected plants (3 dpi).

Figure S7. Selecting candidate EV biomarkers. A) Predicted protein localization of the *Pto* DC3000 EV-enriched proteins whose orthologs are found in *P. aeruginosa* EV proteomes [%] total number of identified proteins is 44 proteins with FDR < 0.005 and EV/WC > 20) in [%]; C = cytosolic; CM = cytoplasmic membrane; E = extracellular; OM = outer membrane; P = periplasm and U = unknown. **B)** Heat map representing transcriptional patterns of the genes coding for EV "core" proteins. Transcriptome data are derived from (33).

Figure S8. Biophysical parameters of *Pto* DC3000 EVs across fractions from gradient enrichment. A) Growth measurements of planktonic *Pto* DC3000 cultures. The yellow arrow indicates EV isolation at the start of early exponential growth stages (0.5-1x 10^9 cfu/mL); the green arrow indicates EV isolation from late exponential growth stages (1.5-2x 10^9 cfu/mL). The dots represent the mean from three biological repeats, error bars represent SD. **B-D)** NTA measurements of particle concentration (A), ζ-potential (B) and size (C) of *Pto* DC3000 EVs collected from each step of gradient enrichment. Different letters indicate significant difference (One-way ANOVA with Tukey post hoc test; $p < 0.05$); no letters indicate no significant differences. The green colour is highlighting the particles from *Pto* DC3000 infected plants (3 dpi).

Table S1. A) Filtered proteomics data with proteins that were identified at least in three from four biological repeats in at least one variant (WC, OM and EV). Values used for volcano plot. Highlighted EV-enriched proteins. **B)** Subcellar localization of identified proteins. **C)** Flagellar proteins identified in EV-enriched proteins. **D)** Expression patterns of genes coding for EV-enriched proteins *in planta.* **E)** Comparisons with *P. aeruginosa* EV proteomes. **F)** Expression patterns of genes coding for EV "core" proteins *in planta* (transcriptome data from (33)).

Table S2. A) GO analysis of EV-enriched proteins by GO class. **B)** GO analysis of EV-enriched proteins cluster I. **C.** GO analysis of EV-enriched proteins cluster II. **C.** GO analysis of EV-enriched proteins cluster III. **D)** GO analysis of EV-enriched proteins cluster IV. **E)** GO analysis of EV-enriched proteins cluster V.

Figure S1

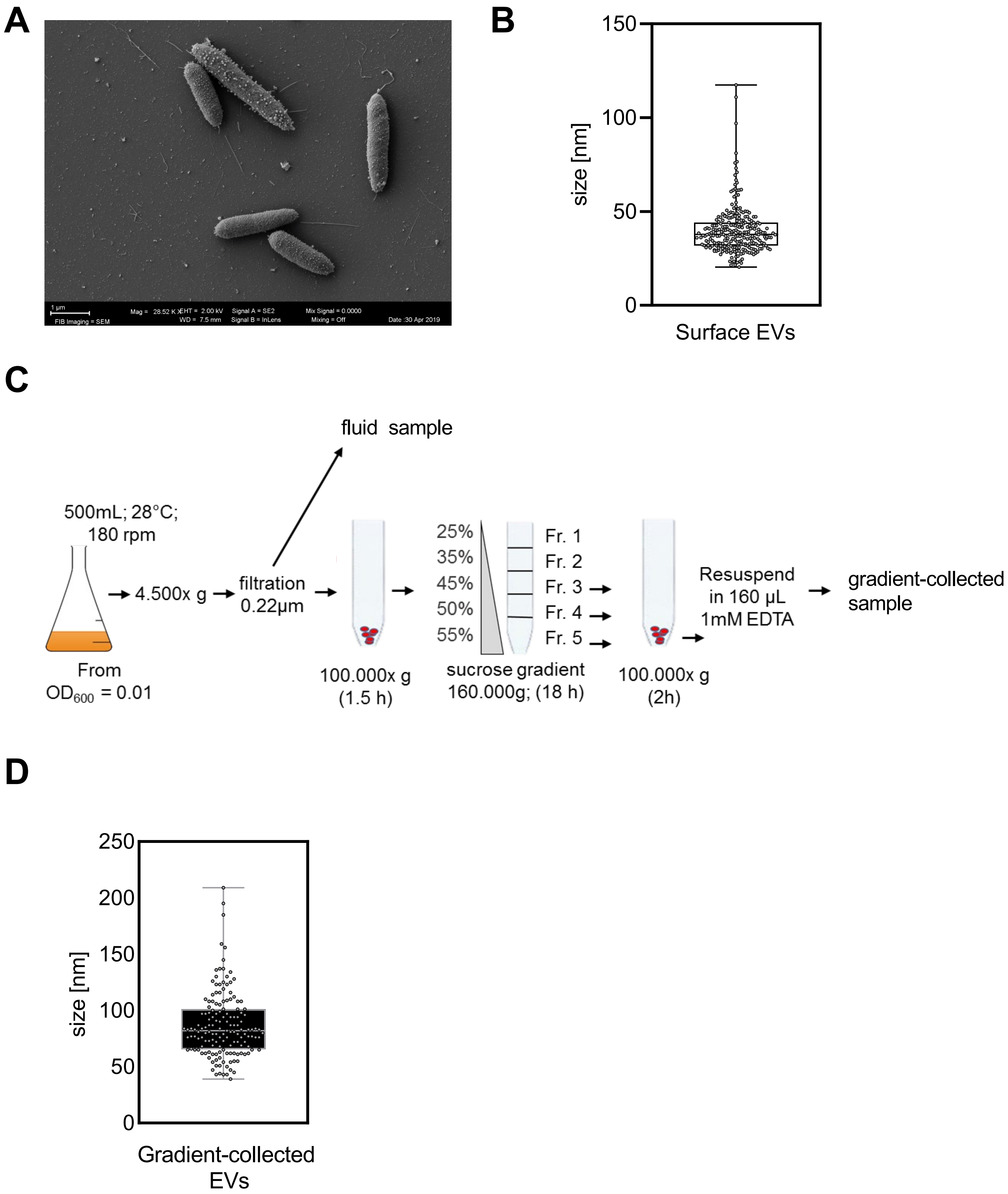
A
1 µm
B
150
100
50
0
size [nm]
Surface EVs
C
fluid sample
500mL; 28°C; 180 rpm
From OD600 = 0.01
4.500x g
filtration 0.22µm
100.000x g (1.5 h)
25%
35%
45%
50%
55%
Fr. 1
Fr. 2
Fr. 3
Fr. 4
Fr. 5
sucrose gradient 160.000g; (18 h)
100.000x g (2h)
Resuspend in 160 µL 1mM EDTA
gradient-collected sample
D
250
200
150
100
50
0
size [nm]
Gradient-collected EVs
