# Supplementary figures and images for: Biophysical and proteomic analyses of Pseudomonas syringae pv. tomato DC3000 extracellular vesicles suggest adaptive functions during plant infection
Source: mBio. 2023 Jun 27;14(4):e03589-22. doi: 10.1128/mbio.03589-22 (PMC10470744; doi:10.1128/mbio.03589-22)

Figure S2

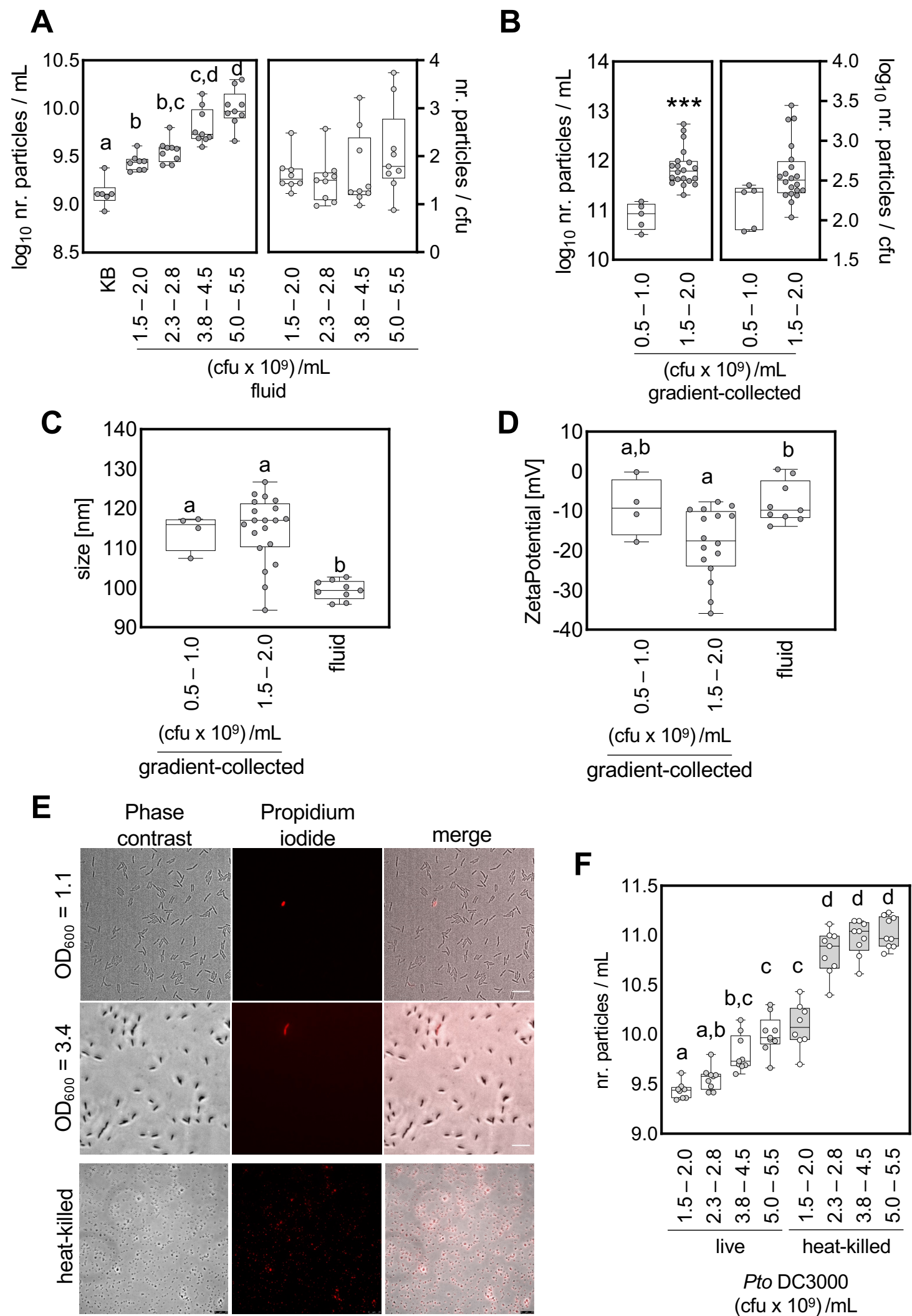

Supplement: Figure S2 — Characteristics of Pto DC3000 EV isolation. [file mbio.03589-22-s0002.pdf]

Figure S3

**A**

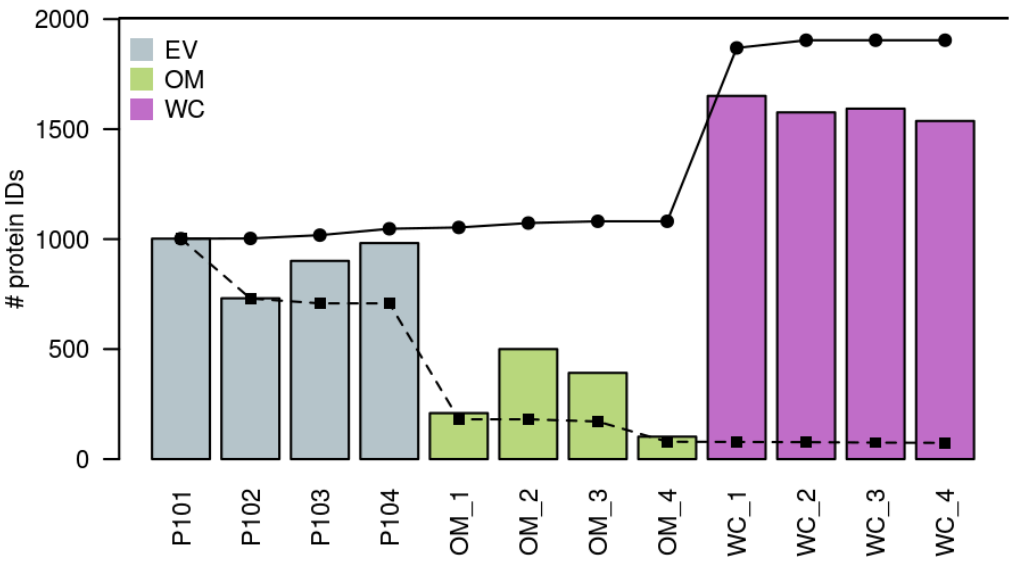

**B**

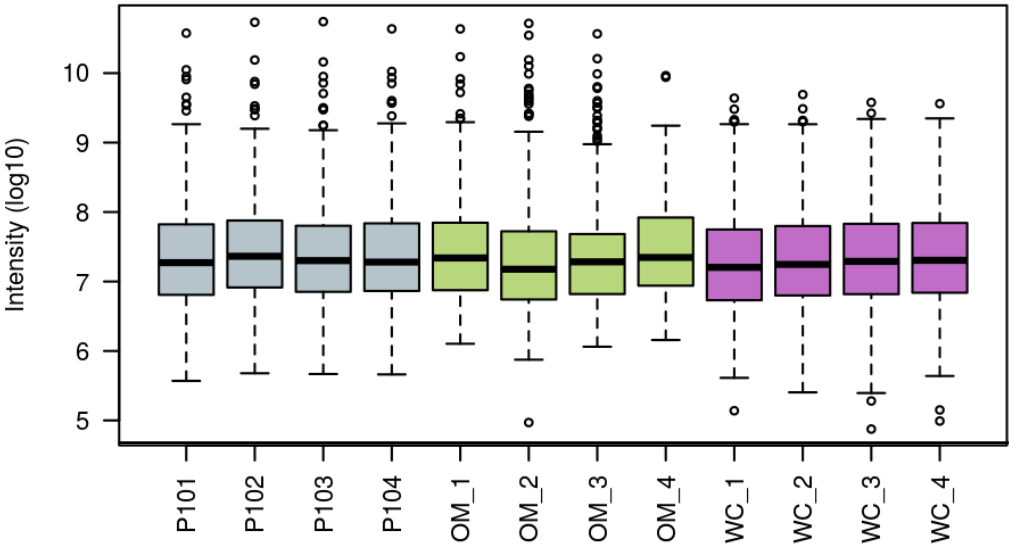

**C**

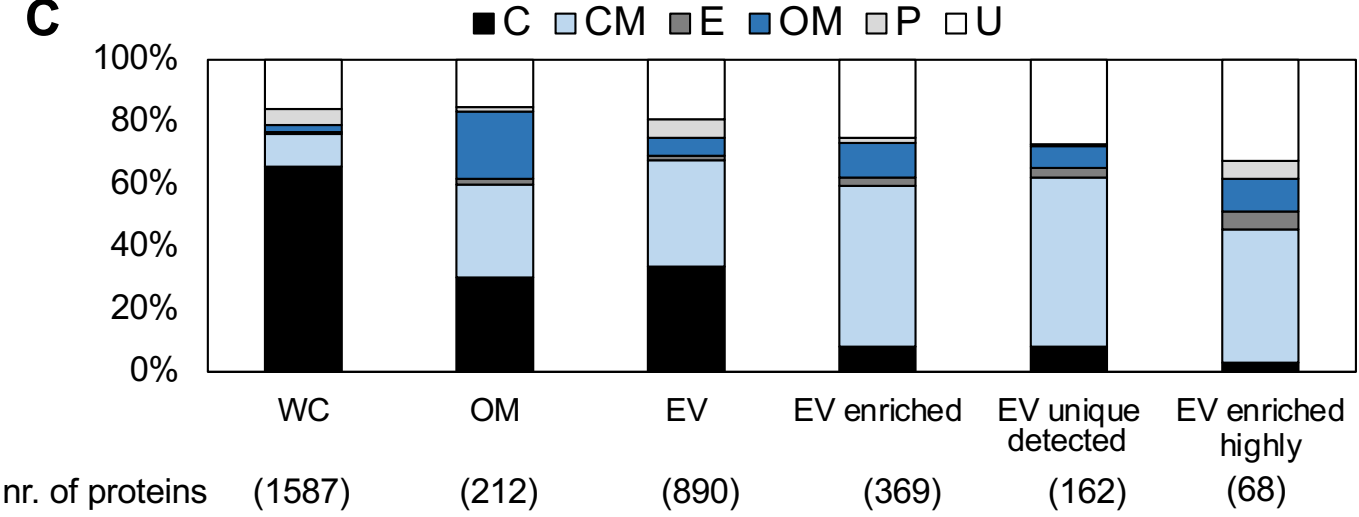

Supplement: Figure S3 — Characteristics of the proteomic analysis. [file mbio.03589-22-s0003.pdf]

Figure S4

**A**

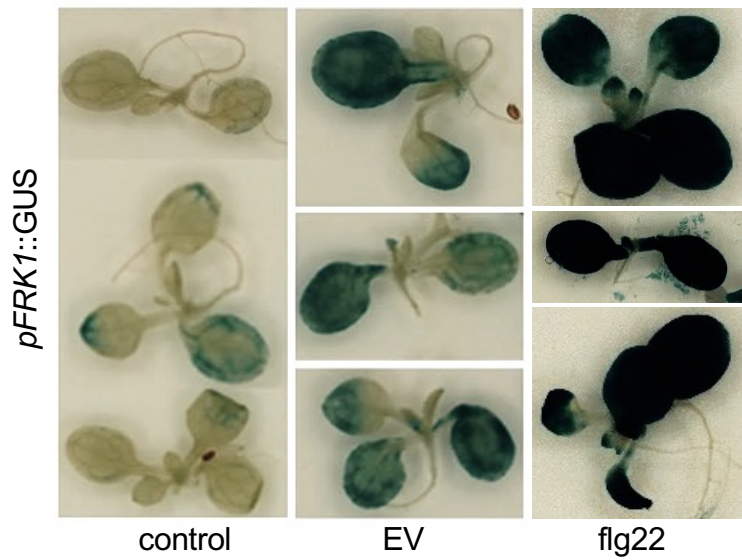

**B**

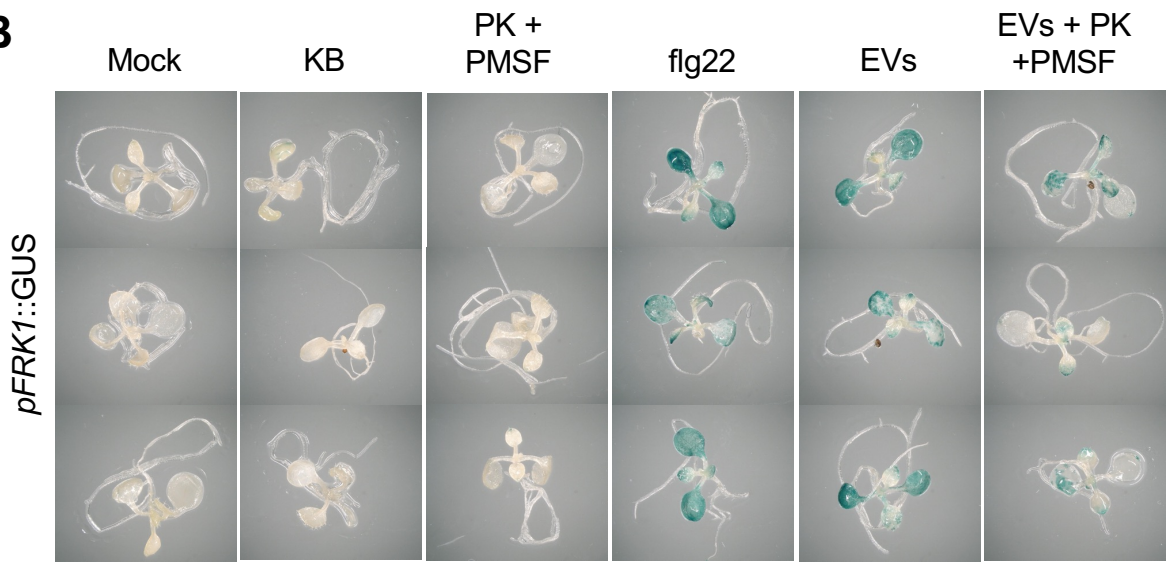

**C**

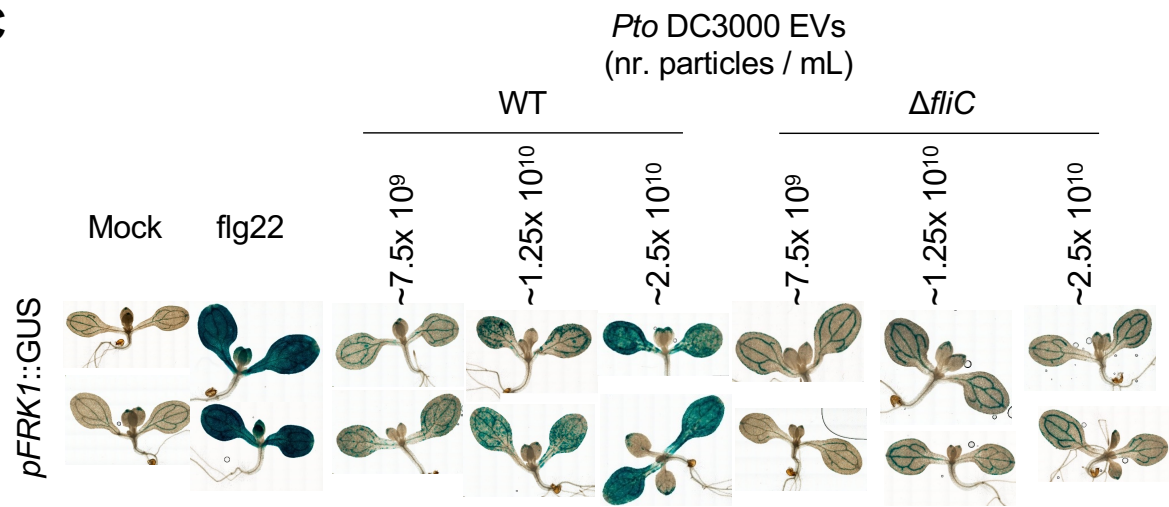

Supplement: Figure S4 — Immunogenicity of Pto DC3000 EVs. [file mbio.03589-22-s0004.pdf]

Figure S5

A

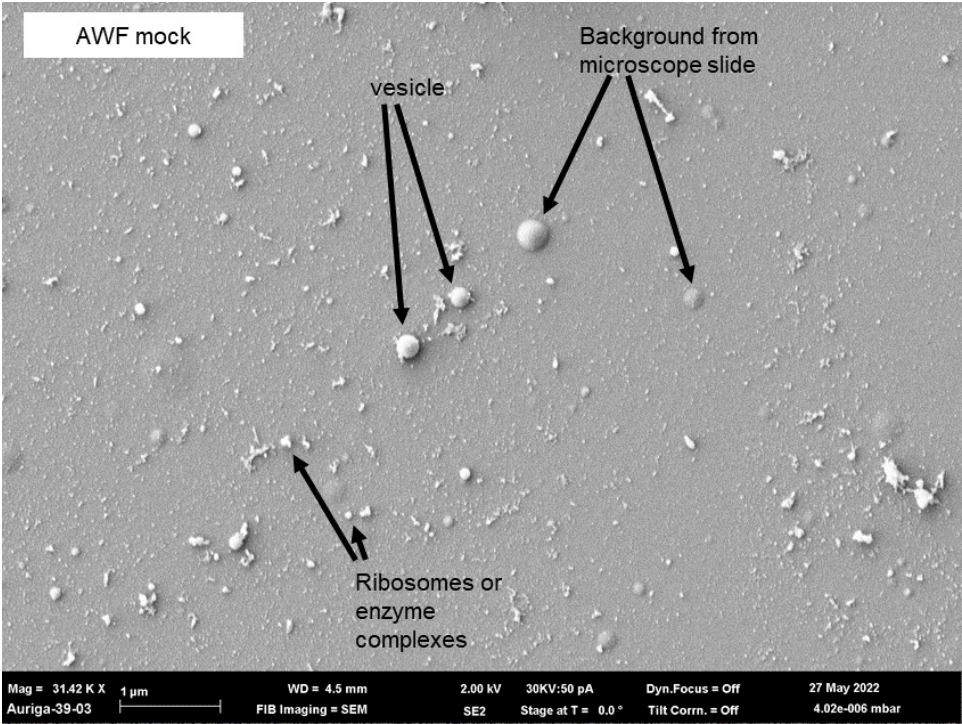

B

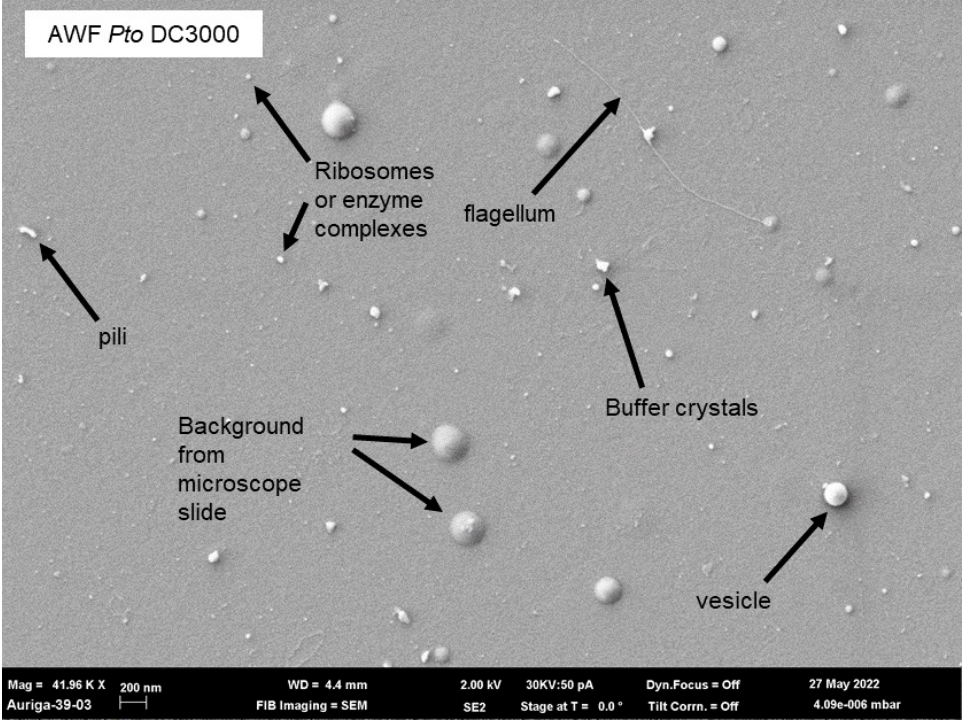

C

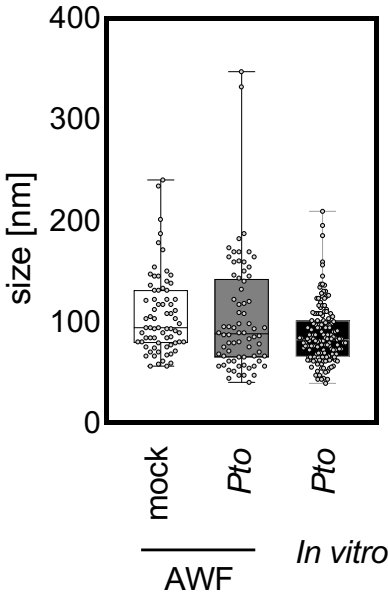

Supplement: Figure S5 — Isolation and observation of vesicles in apoplastic fluids. [file mbio.03589-22-s0005.pdf]

Figure S6

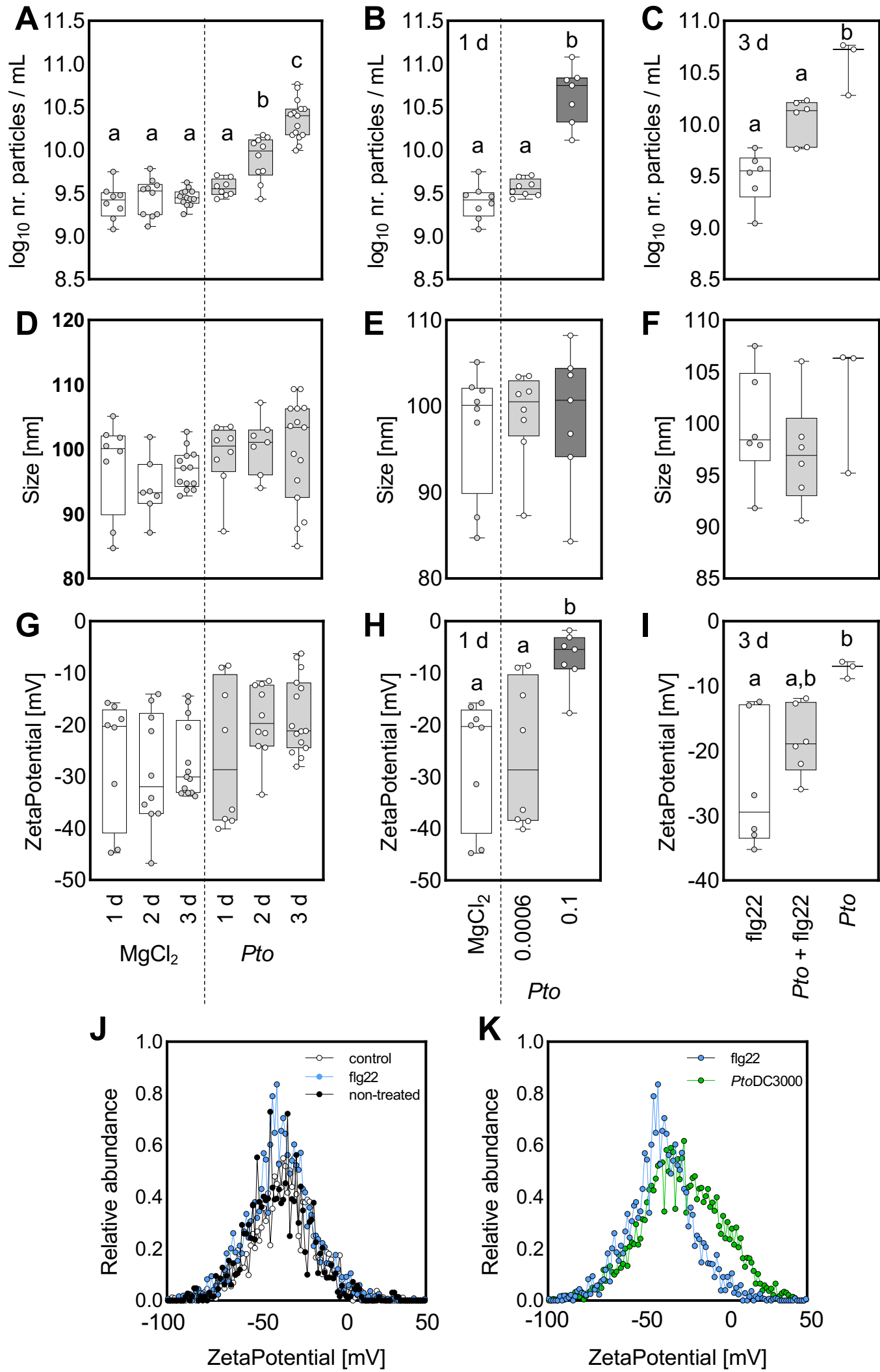

Supplement: Figure S6 — Biophysical parameters of particles in apoplastic fluids from A. thaliana plants infected with Pto DC3000. [file mbio.03589-22-s0006.pdf]

Figure S7

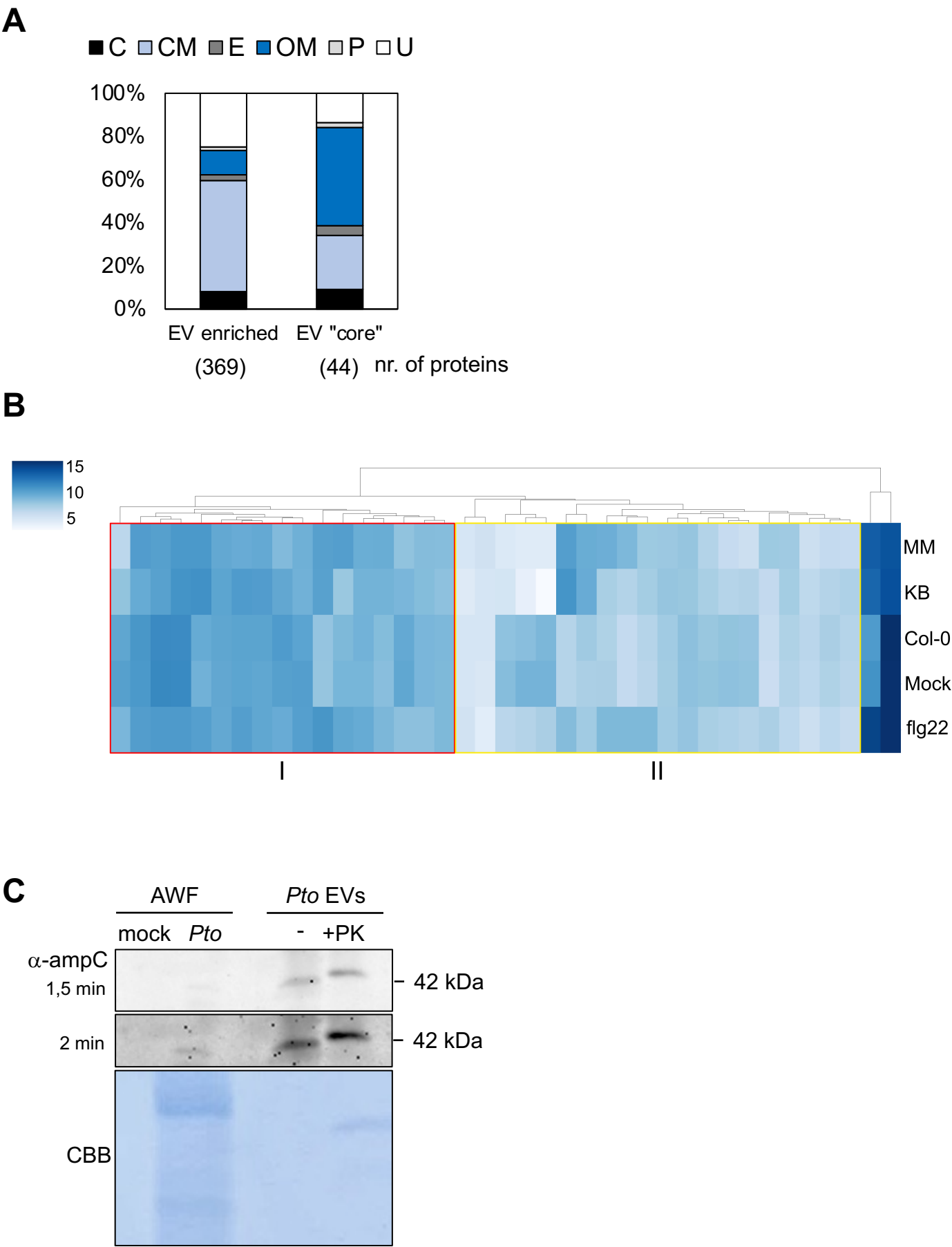

Supplement: Figure S7 — Selecting candidate EV biomarkers. [file mbio.03589-22-s0007.pdf]

Figure S8

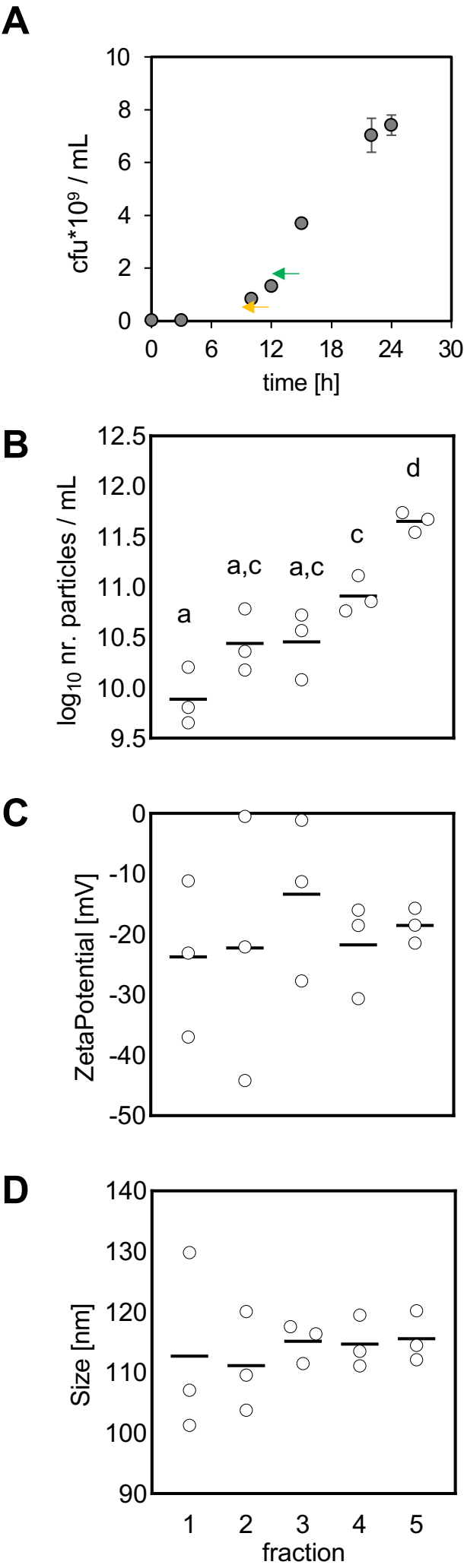

Supplement: Figure S8 — Biophysical parameters of Pto DC3000 EVs across fractions from gradient enrichment. [file mbio.03589-22-s0008.pdf]
